# ICO investors

**DOI:** 10.1007/s11408-020-00366-0

**Published:** 2020-11-06

**Authors:** Rüdiger Fahlenbrach, Marc Frattaroli

**Affiliations:** 1 Swiss Finance Institute @ EPFL, Quartier Unil-Chamberonne, Extranef 211, 1015 Lausanne, Switzerland; 2 ECGI, Brussels, Belgium

**Keywords:** Initial coin offering, FinTech, Individual investors, G20, G24, G32, O33

## Abstract

We conduct a detailed analysis of investors in successful initial coin offerings (ICOs). The average ICO has 4700 contributors. The median participant contributes small amounts and many investors sell their tokens before the underlying product is developed. Large presale investors obtain tokens at a discount and flip part of their allocation shortly after the ICO. ICO contributors lack the protections traditionally afforded to investors in early-stage financing. Nevertheless, returns 9 months after the ICO are positive on average, driven mostly by an increase in the value of the Ethereum cryptocurrency.

## Introduction

In an initial coin offering (ICO), an entrepreneur raises capital by selling a newly minted cryptographic token to the public. The token is usually listed on a specialized exchange quickly after the ICO, creating a secondary market. ICOs have become the prevalent source of financing for start-up companies that use the blockchain technology; more than $30bn have been raised so far through ICOs (Lyandres and Palazzo 2020).^1^ Entities conducting ICOs have unproven business models and are most often in the preproduct stage. There exists virtually no hard information on them, and asymmetric information is large. The financing of such early-stage companies has previously been the domain of highly specialized angel investors or venture capitalists (VCs) who acquire soft information by meeting with potential customers, suppliers and the team and by using sophisticated security design methods guaranteeing priority and control rights. While a significant empirical and theoretical literature on the determinants of post-issue financial success of ICOs has developed, relatively little is known about ICO investors and their reasons to invest. We wish to fill this gap and analyze the composition and trading behavior of the ICO investor base. Most tokens sold in ICOs are “utility” tokens which can be spent to buy a product or service produced by the issuer but do not confer cash flow rights. Our analysis of investor trading behavior seeks to understand whether initial investors primarily buy utility tokens because they are interested in the product (and that therefore, ICOs are a good mechanism for entrepreneurs to understand the market’s demand for the products or platform they develop) or for speculative purposes. We use primary sources (such as ICO whitepapers or an ICO’s *Medium*, *Twitter* and *Telegram* pages as well as the Ethereum blockchain data) to construct a hand-collected sample of successful ICOs with information on the ICO, investors, governance characteristics and products offered, to answer these questions.

The median investor in our sample of ICOs invests only $1200, and each of our sample ICOs has approximately 4700 investors. ICOs therefore appear to have succeeded in tapping a new type of investor to finance innovation, one that security market regulators typically seek to protect. The typical investor makes active use of the secondary market. He sells a substantial fraction of his tokens shortly following the ICO, when the product of the company is not yet developed, indicating that he is more interested in financial gain than the underlying product. Token returns have high variance and positive skewness; both are attributes that retail investors appreciate (e.g., Goetzmann and Kumar 2008 or Kumar 2009). In our sample, investors do not hold a diversified portfolio of ICOs in the same wallet.

A key identifying assumption of our analysis is that ICO investors use one wallet to invest in ICOs and do not camouflage their true investment through multiple wallet strategies. We show through several formal tests that the identifying assumption is defendable for the typical ICO investor. Investors frequently use the same wallet with which they invested into the ICO for other transactions on the Ethereum network afterward, which suggests that they use a wallet for multiple purposes. We show that the value of tokens transferred out of investors’ wallets is highly correlated with trading volume in secondary markets in the same token, implying that most of these tokens are not moved to another wallet belonging to the same investor but rather sold on an exchange. Finally, for ICOs that have a know your customer (KYC) policy, i.e., where the issuer knows the ultimate beneficial owners of tokens bought in the initial sale, the number of contributors disclosed by the issuer after the offering period is statistically indistinguishable from the number of wallets that contributed. The result suggests that most investors invest with one wallet in these ICOs.

ICOs typically happen in two stages. A majority of ICOs holds a closed presale round for larger investors and insiders, during which the participating investors receive a sizeable discount over regular investors. The second phase is the crowdsale stage during which regular investors participate. In our sample, the median discount to presale investors is an economically large 30%. Presale investors can therefore lock in a profit by selling immediately after the ICO if the prevailing secondary market price is at or above the presale price, which is lower than the “list price” paid by regular investors. We find evidence that they do. Large investors sell earlier if there was a presale and if the presale discount was high, and holding period returns to other investors are decreasing in the amount of funding raised in the presale as well as the presale discount. The analysis of the initial participation and subsequent trading patterns by presale investors illustrates a potential issue with the ICO model. Investments by presale investors provide important information to crowdsale investors who interpret the early investments as a signal of the quality of the ICO (e.g., Howell et al. 2019; Fisch 2019), but the possibility of flipping the coins purchased at a discount reduces the information content of presale investor purchases.

We find little evidence that ICO investors receive downside protection or governance rights for their investment, as would be typical for VC or angel investors. Most ICOs do not confer residual cash flow rights to investors, let alone give them liquidation preferences or offer board representation. Only 4% of ICOs specify milestones for the release of funds, and only 4% leave an independent custodian in charge of the funds raised by the company. However, we find some evidence for incentive alignment between investors and entrepreneurs in that a majority of issuers lock up at least part of the tokens held by the issuing firm and its founders. The mean weighted average maturity of the tokens retained by the issuing firm and its founders is 1.1 years.

We conclude with an analysis of secondary market returns. The single most important driver of ICO returns to investors is the concurrent return of Ethereum. Few other variables reliably predict returns 9 months after the ICO. The average gross return (i.e., not adjusted for the returns on Bitcoin or Ethereum) on a token is positive 9 months after the ICO. Average returns in excess of the return of Bitcoin or Ethereum are consistently below unadjusted returns 9 months after the ICO but are, perhaps surprisingly in light of allegations of widespread fraud and pump-and-dump schemes, still positive.^2^

Our paper relates to the literature on the behavior of individual investors (for an overview, see Barber and Odean 2013). In particular, Barber and Odean (2000) document that in their database of retail investors, investors hold on average an undiversified portfolio of only four stocks. Goetzmann and Kumar (2008) show that retail investors hold highly volatile stocks with a high correlation, and Kumar (2009) finds that individuals like to hold stock with high idiosyncratic volatility and skewness. Several researchers have pointed out that investors like to gamble with lottery-like stocks (Dorn et al. 2014; Barber et al. 2009; Gao and Lin 2015; Kumar 2009). The results of these papers are broadly consistent with our findings on ICO investors and can potentially explain the attractiveness of the asset class to retail investors despite the lack of transparency and investor protection.

Our paper is also related to the literature that examines apparently irrational investor behavior in public firms in new industries that promise high growth (e.g., Shiller 2000). Cooper et al. (2001) document that firms that added “.com” to their name during the internet boom experienced abnormal returns of 53% over the following 5 days. Cheng et al. (2019) show that investors react positively to vague 8-K announcements of public firms that they are “going to use blockchain technology in the future.” Lamont and Thaler (2003) demonstrate that investors irrationally bid up prices of equity carve-outs in US technology stocks during the internet boom. Ofek and Richardson (2003) and Lamont and Thaler (2003) suggest that short sale restrictions may explain the persistence of the mispricing of tech stocks during that time. This literature could help explain investor’s appetite for ICOs and the high market valuations, as ICO tokens too are difficult and risky to short.^3^

Our work contributes to an emerging literature on ICOs. Most empirical papers on ICOs relate ICO characteristics collected by secondary sources to measures of ICO success.^4^ Contrary to those papers, we focus on the investors in ICOs instead of the issuers of ICOs. Of the large literature on ICOs, few papers have investigated ICO investors. The only academic analyses of investors in the ICO market so far are—to the best of our knowledge—Howell et al. (2019), Lee et al. (2018) and Boreiko and Risteski (2019). Howell et al. (2019) provide a case study of the investors in the Filecoin ICO, which is interesting but also fairly special because the Filecoin ICO allowed only accredited investors. Lee et al. (2018) use individual investor contribution data to study how quickly the ICO reaches its soft cap and to test the theory of the wisdom of the crowds, and Boreiko and Risteski (2019) analyze investor data to show that only large investors have some ability to time the market and select better ICOs.^5^ Many firms issuing ICOs develop a decentralized trading platform that promises network effects, and much of the emerging theoretical ICO literature has focused on the conditions under which ICOs can create value by solving coordination problems (Bakos and Halaburda 2018; Catalini and Gans 2018; Cong and Li 2020; Li and Mann 2018; Sockin and Xiong 2020). Other theoretical work includes Chod and Lyandres (2020) and Lee and Parlour (2019). The law literature has also started to discuss the legal and regulatory framework for ICOs (e.g., Kaal 2018; Maas 2019; Robinson 2018; Rohr and Wright 2019; Zetzsche et al. 2019).

The remainder of our paper proceeds as follows. Section 2 discusses the data collection procedure. Section 3 presents a brief overview of the ICO market. Section 4 presents the results of our analysis of the characteristics and behavior of ICO contributors. Section 5 contrasts investor protection provisions in venture capital and angel financing with those in ICOs. Section 6 presents regression estimates for whether investor and ICO characteristics matter as determinants of secondary market returns and Sect. 7 concludes.

## Data collection

### Primary market data

We hand-collect data on token sales from primary sources. Our reasons for hand-collecting data are twofold: concerns about data quality and the amount of data items available from secondary sources. Secondary sources often diverge substantially in their assessment of an ICO (see Boreiko and Sahdev 2018; Lyandres and Palazzo 2020 for a systematic analysis of these concerns). Hand collection also allows the inclusion of important characteristics that are not available from secondary sources but are important for our study of ICO investors and investor protection. We collect information on the exact split of funds raised from presale and crowdsale investors, the pricing schedules for both, founder token vesting schedules and whether a venture capitalist has invested into the issuer prior to the ICO. The pricing schedules in particular are important to gain an accurate picture of returns to investors, as discounts given to early and large investors are often sizeable.

To construct our sample, we first create a list of completed ICOs from four secondary sources (icorating.com, smithandcrown.com, icowatchlist.com and coinschedule.com). We retain only records for which the secondary sources indicate that total ICO funding exceeded $1 m. The reason for truncating the sample in this manner is that primary source data on the smaller ICOs are frequently scarce or unavailable. We have compared ICO characteristics of our sample to those of the broad sample used by Lyandres and Palazzo (2020) to understand the representativeness of our sample of ICOs. Our sample of ICOs has approximately the same industry composition, the same variation in state of incorporation, the same fraction with KYC policies and similar number of employees. We find these results reassuring, in the sense that conditioning on fundraising success and size does not meaningfully change the distribution of these general characteristics of the sample.^6^ “Appendix 1” provides the full list of sample ICOs. For the characteristics of those ICOs, we rely exclusively on primary sources such as whitepapers or other documents published by issuers, archived issuer websites kept by the Internet Archive (web.archive.org), company announcements on social media (primarily on *Medium*, *Twitter* and *Telegram*), source code on Github, company announcements on the bitcointalk.org message boards and various national commercial registers. To make sure we always use the original version of whitepapers and other documentation available during the fundraising, we used the Internet Archive’s Wayback Machine (web.archive.org) to retrieve the last version of the whitepaper published before the ICO. We were able to retrieve this version for the vast majority of ICOs. Furthermore, we sometimes consult the *Crunchbase* database for information on venture funding. “Appendix 2” defines all collected attributes in detail.

Our final sample consists of 306 ICOs that collectively raised over $6.2b in funding between March 2016 and March 2018.^7^ In 2017 alone, they raised $5 billion.^8^

### Secondary market data

We retrieve secondary market prices in US dollars from coinmarketcap.com. The webpage aggregates traded prices from all cryptocurrency exchanges that provide data on prices and trading volumes through a public application programming interface and then calculates volume weighted average daily open, high, low and closing prices. We observe secondary market prices for 276 out of 306 sample ICOs (90%).

We calculate continuously compounded returns in US dollars based on the average price paid by crowdsale investors.^9^ Where the average price is unavailable (which is the case for 24% of ICOs), we base returns on the mid-price, i.e., the average between the highest and the lowest price paid by investors in the crowdsale. We use continuous compounding because most ICOs trade continuously.

### Ethereum blockchain data

Over 90% of our sample ICOs sell crypto tokens hosted on an existing blockchain, most commonly Ethereum. The publicly available Ethereum data enable us to provide statistics such as the median contribution per wallet (we use the terms address and wallet interchangeably) and the number of sample ICOs to which each wallet contributes. We can also follow the issued tokens through time and analyze how quickly investors sell their tokens.

All data we observe only identify parties by their Ethereum address, and multiple Ethereum addresses belonging to the same person or organization cannot be easily reconciled. The main assumption underlying our investor analysis is that the representative ICO investor only controls a single Ethereum address and that we can equate wallets with investors. We believe and provide several formal pieces of evidence in Sect. 4 that our main assumption can be maintained for many investors.

An Ethereum account consists of a public key, part of which (after a mathematical transformation called hashing) forms an *address,* representing the equivalent of a bank account number to which transactions can be sent. A corresponding private key (the equivalent of a password) controls transfers from the account. All transactions and token transfers made between different addresses on the Ethereum blockchain are publicly available and downloadable.^10^ Ethereum addresses can either be controlled by a human being or a smart contract. The latter is a piece of computer code that interacts with other parties on the Ethereum network according to a set of rules. The ERC20 contract is a popular smart contract for ICOs that contains a ledger that tracks the number of tokens held by each address. When tokens are sold or spent, the ledger is modified to reflect their new owner. Every change in token ownership requires interacting with the token contract to change the ledger.

**Fig. 1:**
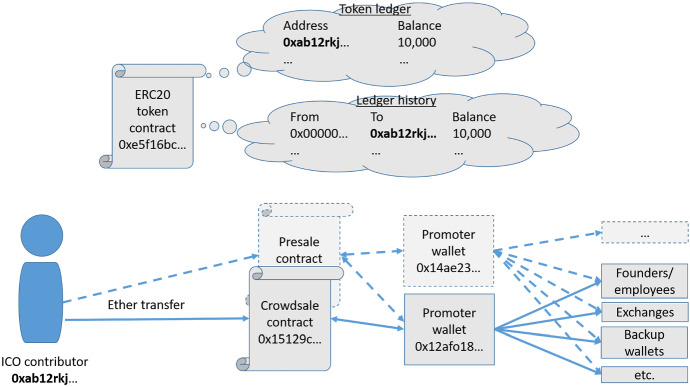
Illustration of contribution flows during an ICO on the Ethereum platform

During an initial coin offering on Ethereum, contributors send Ether to an address controlled by the promoter (the “token sale address”) with the promise of being allocated tokens in an ERC20 contract in return. Deriving comprehensive information on the investor base from the transactions associated with contributions is typically not possible because of a number of challenges, which are visualized in Fig. 1. The presale and crowdsale stages usually use different contracts and transactions made toward the token sale address are not always limited to ICO contributions (the promoter will usually send some Ether to the address to pay for transaction costs, for example). Because the presale stage is usually private, the Ethereum address used during the presale is often not public knowledge. In addition, contributions made using means of payment other than Ether (e.g., US dollars or Bitcoin) will not show up as transactions on the blockchain. We therefore decided not to analyze the *contributions* made by investors, but instead focus on the *distribution* of tokens to investors following the ICO. Knowing the token prices from our manually collected dataset, we can infer the approximate investment per Ethereum address from the number of tokens allocated following the ICO.

ICO promoters can distribute tokens in two ways. The initial balance can be allocated to the crowdsale contract or one or more addresses controlled by the ICO’s promoter, from which the tokens are then reallocated to contributors. In that case, we observe one or more ERC20 token transfers from the initial address to the contributor’s address. Alternatively, the token can be made *mintable,* in which case there is no initial balance but tokens are “created” from nothing for every contributor. In that case, we observe a token transfer from the “zero address” to the contributor.

We generally do not know from which address the initial token distribution is made. We address this challenge by analyzing the first 100 transfers made for each token in the sample. If at least 98 of them have the same source, we assume that the most common source within those 100 transactions is the unique address from which token distributions originate.^11^ Second, some transfers are not made in exchange for a financial contribution but represent an allocation to the founding team or free, promotional distributions to the general public (“air drops”) to publicize the new token. We exclude transfers where the amount of tokens sent is worth less than 50 USD or where the receiving address receives more than 10% of the total token supply in all transactions to avoid such token transfers contaminating our sample. The Ethereum platform hosts 264 out of the 306 sample ICOs. We are able to identify the token contract address and the token transfers for 247 of those ICOs. We further know the average price or average crowdsale price paid by investors for a subset of 181 and unambiguously identify the Ethereum address from which the initial token allocation occurred for 98 of those ICOs. These 98 ICOs received over $2.3b in funding and represent about a third of all money raised in our total sample. From now, we will call this subsample of our data “the investor sample.”

## Description of the ICO market

We briefly describe the typical structure of an initial coin offering and summarize the characteristics that are important for our subsequent analysis in Table 1.^12^ In an ICO, an issuer sells a newly minted *cryptocurrency* or *cryptographic token* to the public. The ICO ends once the contribution period is over or once it reaches the maximum amount of funding (if applicable). A decentralized ledger (blockchain) tracks token ownership thereafter and tokens trade in secondary markets shortly following the ICO. The ICO can either be based on a new, standalone blockchain ledger or be implemented as a *smart contract* on an existing platform (which is the case for 91% of the sample). The Ethereum platform typically hosts the cryptographic tokens.^13^ The majority of firms in our sample of successful ICOs raised between $1 m and $40 m through their ICO. Often, the ICO comprises two stages. In our sample, 68% of ICOs begin with a *presale* (also known as *pre*-*ICO* or *private sale*) stage, in which larger investors can purchase tokens at discounted prices. In a subsequent *crowdsale* (also known as *public sale*) stage, the general public can acquire tokens. The mean ICO received $24.2 m over all rounds and $18.0 m during the crowdsale stage. Hence, the crowdsale investors contribute the majority of funds.

**Table 1:** Descriptive statistics

	Mean	Median	Min	Max	SD	*N*
*Panel A: ICO characteristics*						
Is cryptographic token	0.91	1.00	0.00	1.00	0.29	306
Has a presale	0.68	1.00	0.00	1.00	0.47	306
Total amount raised (USDm)	24.16	15.07	1.01	233.00	33.16	228
Amount raised in crowdsale (USDm)	18.03	10.76	0.50	218.84	26.70	262
Amount raised in presale (USDm)	6.02	1.12	0.00	193.65	15.01	246
Fundraiser has minimum (“soft cap”)	0.44	0.00	0.00	1.00	0.50	306
Fundraiser has maximum (“hard cap”)	0.95	1.00	0.00	1.00	0.22	306
Percentage of hard cap raised (%)	70.16	81.39	2.34	180.65	38.89	204
Presale discount (%)	34.18	30.00	− 16.50	96.88	23.17	152
Crowdsale max. discount (%)	17.36	15.00	0.00	98.57	18.76	288
Token share crowdsale investors (ex ante)	0.47	0.49	0.01	1.00	0.27	248
Token share presale investors (ex ante)	0.11	0.04	0.00	0.70	0.15	247
Token share team (ex ante)	0.39	0.38	0.00	0.96	0.22	292
Token share producers/miners (ex ante)	0.02	0.00	0.00	0.88	0.12	300
Unsold tokens “burnt” or proportional alloc.	0.55	1.00	0.00	1.00	0.50	306
Product or prototype developed	0.51	1.00	0.00	1.00	0.50	306
Qualified investors only	0.03	0.00	0.00	1.00	0.18	306
US retail investors excluded	0.51	1.00	0.00	1.00	0.50	306
High-quality advisory team	0.41	0.00	0.00	1.00	0.49	306
Use of proceeds mentioned	0.71	1.00	0.00	1.00	0.45	306
Legal advisor disclosed	0.25	0.00	0.00	1.00	0.44	306
Has VC backing	0.26	0.00	0.00	1.00	0.44	306
KYC/AML procedure	0.48	0.00	0.00	1.00	0.50	306
*Panel B: Investor protection*						
Is a security	0.22	0.00	0.00	1.00	0.41	306
Legal form and jurisdiction known	0.88	1.00	0.00	1.00	0.33	306
Legal entity is corporation or LLC	0.90	1.00	0.00	1.00	0.31	269
Registered in offshore financial center	0.20	0.00	0.00	1.00	0.40	306
Funding milestones	0.04	0.00	0.00	1.00	0.20	306
Independent custodian for ICO funds	0.04	0.00	0.00	1.00	0.19	306
Team tokens locked up	0.58	1.00	0.00	1.00	0.49	306
Team lockup period (weighted avg.)	1.10	0.75	0.02	5.50	0.99	179
Presale tokens locked up	0.14	0.00	0.00	1.00	0.34	207
Presale lockup period (weighted avg.)	0.53	0.27	0.02	2.00	0.52	28
Investors have governance rights	0.18	0.00	0.00	1.00	0.38	306

The table shows summary statistics for a hand-collected sample of 306 ICOs that took place between March 2016 and March 2018 and raised at least $1 m according to secondary sources. All variables are defined in “Appendix 2”

ICOs frequently have a *soft cap* (45%) and/or a *hard cap* (95%). If the ICO contributions do not reach the soft cap, the company returns funds to the sender (ensured by an escrow arrangement or smart contract). The soft cap is therefore similar to the threshold model applied by popular crowdfunding websites such as Kickstarter (see, e.g., Mollick 2014). The hard cap is the maximum amount of funding the issuer will accept. On average, sample ICOs raised 70.2% of their hard cap, including the presale stage.

It is rare that all investors pay the same price for the tokens. The presale usually takes place at heavily discounted prices, and early and/or large investors in the crowdsale obtain a discount as well. On average, presale investors receive a 34% discount over the “list price,” whereas the earliest (or largest) crowdsale investors receive a 17% discount.^14^ The issuer on average offers 47% of the total token supply for sale during the crowdsale. Presale investors hold an average of 11% of the anticipated post-ICO token supply as of the time of the crowdsale, while the founders hold 39%. On average, a mere 2% of tokens are reserved for miners (the parties carrying out the verification of transactions on the blockchain), reflecting that most ICOs issue non-mineable tokens on the Ethereum blockchain. More than half (55%) of ICO issuers destroy unsold tokens after the offering period. Only 51% of ICO issuers have a product or prototype. A minority of ICO promoters has decided to avoid securities regulations by only offering tokens to accredited or qualified investors (3%), or only to foreign investors and accredited US investors (51%). Such restrictions remove an important advantage of an ICO: to gauge demand for the product by future users. Issuers often disclose their advisory team, 41% of which we judge to be “high-quality” advisory teams consisting of venture capitalists, researchers, executives and entrepreneurs. In general, the level of disclosure varies substantially in the cross section; 29% of ICOs do not even disclose their intended use of the money raised (e.g., by category of expenses), and 25% of issuers disclose the name of the legal advisor that assists them with the transaction to the public. At the time of the ICO, 26% of issuers have received VC funding.

ICO tokens can help launder money gained in illicit ways. To comply with anti-money-laundering legislation, 48% of sample ICOs have adopted AML (anti-money-laundering) or KYC procedures, which verify the identity of an investor before accepting an investment. The awareness of regulatory issues has been increasing among ICO issuers. The fraction of ICOs with a KYC policy has been steadily increasing, from 0% in the first quarter of 2017, to 80% during the first quarter of 2018.

Panel B of Table 1 describes characteristics related to investor protection. The fraction of security token (i.e., tokens for which the issuer promises to make payments to their owner in the future) in the sample is 22% but has been falling, from a high of 40% during the first quarter of 2017 to only 14% a year later. We were able to identify the jurisdiction and legal form for 88% of all entities organizing ICOs using the material provided by the issuer and publicly searchable commercial registers. Among the identifiable subset, 90% are either joint-stock or limited liability companies (or their international equivalents), i.e., entities typically associated with for-profit commercial activity. Offshore financial centers, using the definition of the International Monetary Fund (IMF), host 20% of all ICOs. Only 4% of ICOs specify milestones for the release of funds and 4% specify an independent custodian for the funds raised. A majority (58%) of ICOs implement vesting periods for the tokens allocated to the company and its founders. The weighted average vesting period for locked up tokens is 1.10 years. Only 14% of ICOs specify a lockup period for tokens owned by presale investors, on the other hand. Those that do lock them up for 0.53 years on average. Only 18% of ICOs give investors governance rights, usually by allowing them to vote on certain topics.

## Analysis of ICO investors

We now turn to the main analysis of the characteristics and trading patterns of ICO investors, using the investor sample. In Sect. 4.1 we first address the central question of whether our key identifying assumption that we can approximately equate the number of cryptographic wallets holding a token with the number of investors in an ICO is defendable. In Sect. 4.2, we provide evidence that an aggregation of all distributed coins multiplied with the price per coin from our Ethereum data approximately equals the total amount of funds raised during the ICO. We also show summary statistics along several key ICO characteristics for the investor sample and compare it to the overall sample to analyze how different the investor sample is from the overall sample. Section 4.3 then analyzes the average contribution size, Sect. 4.4 examines the determinants of investor participation in the crowd sale, and Sect. 4.5 analyzes the fraction of repeat contributors. Finally, Sect. 4.5.1 attempts to identify crowdsale and presale investors’ motivation for participating in ICOs.

### Is the assumption that the typical investor invests with only one address per ICO defendable?

Investors can open wallets at no costs (although it is costly to send funds and tokens from one Ethereum wallet to another even if they have the same owner) and wallets are pseudonymous, i.e., it is impossible for a researcher to link wallets to identities. Throughout the analysis in Sect. 4, we equate wallets with investors. Investors may want to use multiple wallets for at least two reasons. They may want to hide from the issuing firm that they are a large investor or they may want to hide this information from the general public. One potential concern with our analysis is that we overestimate the number of investors and underestimate the contribution amount because investors use multiple wallets for the same ICO. A second concern relates to our analysis of investor trading behavior post-ICO. We may overestimate the trading activity of ICO investors, if investors move tokens from one of their wallets to another one.

We conduct several tests to reduce concerns about our main assumption. Our first piece of evidence comes from a comparison of movements of tokens out of ICO investors’ wallets with trading volume for that token on cryptocurrency exchanges. This test seeks to establish that the majority of investors who move tokens out of their wallet do so to sell them on an exchange rather than to move them to another of their own wallets. If a significant number of original ICO investors did not sell their tokens post-ICO, but rather moved them from one of their wallets to another, exchange-reported trading volume on a given day would not correlate highly with changes in the tokens held by the wallets participating in the ICO. The correlation between exchange-reported trading volume and our implied (from Ethereum) sales by ICO investors is, however, very strong. We calculate daily implied sales for the first 90 days after the ICO as the gross number of tokens moved out of ICO investors’ wallets multiplied with the average between the daily opening and closing price. We aggregate implied sales by ERC20 token and day. We then estimate a regression of the actual daily trading volume reported by coinmarketcap on daily implied sales by ICO investors (having winsorized both at the 1% and 99% levels) and time and token fixed effects. The coefficient on implied sales is 0.92 (*t* = 9.30), so for every USD in implied sales the actual volume increases by 0.92 USD. Hence, when the token balance of an ICO investor drops, the tokens are most often traded on an exchange and not moved to a different wallet of the same investor.

Second, we also examine how often addresses are used for sending and receiving Ether following their investment in an ICO. If investors created a new wallet for every ICO, it is unlikely that they would frequently be using these special-purpose wallets for transactions afterward. We find that in the first 270 days following a contribution to an ICO, the median address is used for two transactions, outgoing or incoming, with a total volume of $210.11 valued at the Ether prices of those dates. We interpret this number as evidence that investors use the wallets with which they participate in ICOs also for other purposes. Note that the total volume we analyze would only include proceeds from the sale of ERC20 tokens if the investor explicitly transferred the sales proceeds from their exchange account to the same Ethereum wallet. In addition, the total volume is also larger than what investors would typically keep in their wallet to pay for transaction cost.^15^ Our third and final set of tests relies on the existence of a KYC policy at the ICO. If an ICO has a KYC policy, investors have no incentive to use multiple addresses to hide their identity from the issuing firm (although they may still do so to hide their identity from the public). Our first test uses the existence of a KYC policy together with the voluntary disclosure of the number of contributors to the offering by some issuing firms. Because these firms know the individuals associated with each address, their self-reported number of contributors should reflect the actual number of investors rather than the number of contributing addresses. In particular, if many ICO participants use multiple wallets to hide their true investments, the number of self-reported contributors should be much lower than the number of wallets that we identify. Using a simple *t* test, we find that for ICOs with a KYC policy, the self-reported number of contributors actually slightly exceeds our estimate for the number of investors, but insignificantly so.^16^ The result means it is unlikely that a large fraction of investors are using multiple wallets to hide their identity from the public; if they did, our estimate for the number of investors would significantly exceed the self-reported number in this subsample. We also test whether our estimate for the number of contributors for ICOs with a KYC policy is different from our estimate for the subset without one. If investors systematically use multiple wallets to hide their identity from the issuer, our estimate for the number of contributors should be higher for those ICOs that do not have a KYC policy than for those that do. However, a two-sample *t* test indicates that our estimate for the number of contributors for ICOs with a KYC policy actually exceeds that of ICOs without one, with marginal statistical significance.^17^ Therefore, we do not find any evidence indicating that investors are systematically using multiple addresses to hide their identity from the issuing firm.

### Data quality and representativeness of the investor sample

Table 2 compares the actual amount of funding and the amount implied by our analysis of token distributions for the investor sample. The mean of the implied amount of funding is $26.0 m and is statistically indistinguishable from the mean of the actual amount, which is $23.1 m. The medians are similarly close but reversed in order, with $12.7 m for the implied total and $14.6 for the actual. Some ICOs also disclose the number of unique contributors. We collect such disclosures for the investor sample and compare them to the number of contributors derived from our analysis in Panel B of Table 2. The two means are statistically indistinguishable.

**Table 2:** Comparing disclosed and calculated amounts of funding and number of contributors

	Mean	Median	Min	Max	SD	Obs.
*Panel A: Funding*						
Total amount raised (USDm)	23.12	14.57	1.25	159.28	30.15	74
Implied total calculated (USDm)	26.01	12.72	0.13	240.92	41.02	74
*t* *test for difference in means*	*1.50*	*p*-*value*	*0.14*			
*Panel B: Number of contributors*						
Self-reported number of contributors	4687.94	2950.00	500.00	25,000.00	4970.68	32
Implied number of contributors calculated	4220.53	1698.00	505.00	21,297.00	4713.63	32
*t* *test for difference in means*	*1.24*	*p*-*value*	*0.22*			

The table compares the actual amount of funding and the number of contributors with the corresponding amounts implied by our analysis of token distributions for 98 ICOs conducted on the Ethereum blockchain (the “investor sample”). We exclude ICOs for which we cannot identify with certainty the Ethereum address from which the tokens have been initially distributed. Furthermore, transfers where the amount transferred is worth less than 50 USD or where the receiving address holds more than 10% of the total token supply are excluded. Contribution amounts are only calculated for ICOs where the average prices for presale and crowdsale are less than 50% apart. The implied total is calculated as the mean US dollar contribution per ICO participant times the number of participants implied by token distributions following the ICO

Table 3 compares the investor sample to the remaining ICOs based on several characteristics. The two samples differ along two dimensions: the fraction of security tokens and the fraction of ICOs with a KYC procedure. 59.2% of ICOs in the investor sample have KYC verification against 42.8% of the remaining ICOs. Similarly, only 14.3% of tokens in the investor sample are unambiguously securities, compared to 25.0% of the remaining ICOs. Importantly, ICOs in the investor sample are not any more or less likely to restrict participation by retail investors. Based on these results, we conclude that there is sufficient overlap in characteristics between the two subsamples and that the investor sample is representative of the typical ICO in our overall sample.

**Table 3:** Descriptive statistics for the “investor sample” and other ICOs

	Investor sample	Other ICOs	Difference
Total amount raised (USDm)	23.185 (27.14)	21.617 (32.94)	− 1.568 (3.67)
Amount raised in presale (USDm)	6.130 (9.92)	5.961 (17.34)	− 0.168 (1.74)
Has VC backing	0.245 (0.43)	0.274 (0.45)	0.029 (0.05)
US retail investors excluded	0.582 (0.50)	0.476 (0.50)	− 0.106* (0.06)
Qualified investors only	0.020 (0.14)	0.038 (0.19)	0.018 (0.02)
Registered in offshore financial center	0.173 (0.38)	0.216 (0.41)	0.043 (0.05)
Is a security	0.143 (0.35)	0.250 (0.43)	0.107** (0.05)
KYC/AML procedure	0.592 (0.49)	0.428 (0.50)	− 0.164*** (0.06)
Investors have governance rights	0.204 (0.41)	0.168 (0.38)	− 0.036 (0.05)
Observations	98	208	306

The table compares the means of select attributes for the subsample of 98 ICOs conducted on the Ethereum blockchain for which we can calculate descriptive statistics for investors’ contributions with those of all other sample ICOs. All variables are defined in “Appendix 2.” Parentheses in the first two columns contain standard deviations. The third column displays the difference in means and, in parentheses, the associated standard error. One, two and three asterisks indicate statistical significance at the 10, 5 and 1% level, respectively

### Average contribution size

We analyze the contribution per investor in Table 4. The mean of the median contribution per investor is $1203.35. The small dollar amount suggests that the majority of investors are not like the accredited investors that would typically participate in angel financing rounds.^18^ Hellmann et al. (2017) for example examine data from British Columbia’s Investment Capital Program and find that Canadian angel investors invest on average $440’000 in first rounds. Goldfarb et al. (2014) examine data on 182 Series A US financings and find that the mean investment by an angel investor is $150,375, while the median investment size is $25,000. Additional evidence for the frequent participation of retail investors in ICOs comes from the average number of investors, which at 4698.91 is three orders of magnitudes larger than the number of investors in a typical angel financing round. The number of ICO contributors and the amount of financing per contributor also significantly exceed the number of backers and contributed amounts in the average successful Kickstarter crowdfunding project. Mollick (2014) uses the universe of Kickstarter projects from its inception in 2009 to July 2012. He estimates that on average 122 individuals contribute $80.55 each to a typical Kickstarter project in his sample. The data suggest that ICO promoters tapped a new type of start-up investor.

**Table 4:** Contribution amount per address in Ethereum ICOs

	Mean	Median	Min	Max	SD	*N*
Minimum contribution (USD)	65.00	50.60	50.00	464.86	65.31	74
Maximum contribution (USDm)	3.23	1.05	0.00	37.11	6.10	74
Mean contribution (USD)	10,093.88	4355.20	809.42	128,301.83	17,516.03	74
Median contribution (USD)	1203.35	697.95	158.95	13,976.73	1965.85	74
SD of contribution (USD)	87,907.93	41,707.62	1524.86	1,025,779.56	153,958.89	74
Number of contributors	4698.91	2312.50	81.00	39,356.00	6672.74	98

The table displays summary statistics for the ICO contributions made per address on the Ethereum platform. US dollar amounts are calculated as the number of tokens transferred to the investor times the average price per token over the entire ICO, including the presale. We exclude ICOs for which we cannot identify with certainty from which Ethereum address the tokens have been initially distributed. Furthermore, transfers where the amount transferred is worth less than 50 USD or where the receiving address holds more than 10% of the total token supply are excluded. Contribution amounts are based on the average price over both presale and crowdsale only calculated for ICOs where the average prices for presale and crowdsale are less than 50% apart

The skewness of the ICO contribution amount distribution is positive, with the mean of the average contribution per investor amounting to $10,093.88, suggesting that a small number of larger investors exist, with contributions likely often made during the presale.^19^

### Determinants of investor participation in the crowdsale

Next, we ask whether retail investors are drawn to ICOs with certain characteristics. For this purpose, we regress our estimate for the (natural logarithm of the) number of contributors on ICO characteristics.

Ex ante, we expect that the number of investors will be increasing in the level of disclosure, the number of investor protections and the presence of presale investors and venture capitalists that might fulfill a monitoring or certification function for the ICO. We therefore include these characteristics in the regression. We also control for an ex ante measure of size (the hard cap) and several core ICO attributes such as whether the issuer has developed a product or prototype, whether it is advised by a high-quality advisory team, and whether there is a KYC procedure. These variables provide a proxy for the quality of the ICO and its demand for funding. In addition, the tests contain month-year fixed effects based on the first day of the ICO.

The regression results are presented in Table 5. In the first column, we include all ICOs and control for the existence of a presale through an indicator variable. In column 2, we condition on the ICO having had had a presale and include a control for the natural logarithm of the amount of money raised in the presale. Column 1 shows that ICOs with a presale attract 87.8% more investors, statistically significant at the 5% level. (The dependent variable is log-transformed, the marginal effect of having a presale is therefore exp(0.63) − 1 = 0.878). However, an increase in the amount raised in the presale does not have a significant impact on the number of investors (column 2). Most of the variables describing ICO attributes and disclosure are insignificant as well. An exception is the existence of a KYC policy, which is associated with a 74.5% increase in the number of investors.

**Table 5:** Determinants of the number of contributors

	(1)	(2)	(3)	(4)	(5)	(6)
Has a presale	0.630** (2.22)		0.741** (2.24)		0.563 (1.28)	
Ln(presale amount) (USDm)		0.040 (0.20)		0.028 (0.14)		− 0.130 (− 0.43)
Use of proceeds mentioned	− 0.251 (− 0.99)	0.123 (0.34)	− 0.334 (− 1.17)	− 0.247 (− 0.51)	− 0.199 (− 0.73)	− 0.050 (− 0.07)
Offshore incorporation	0.229 (0.70)	− 0.319 (− 0.50)	0.204 (0.56)	− 0.463 (− 0.84)	1.721^**^ (2.35)	2.196 (1.40)
Legal form and jurisdiction known	− 0.061 (− 0.12)	− 1.251 (− 1.35)	− 0.003 (0.56)	− 1.119 (− 1.24)	− 0.700 (− 1.02)	− 1.996^*^ (− 2.07)
Legal advisor disclosed	0.018 (0.06)	− 0.052 (− 0.12)	0.013 (0.04)	− 0.351 (− 0.66)	0.059 (0.19)	− 0.116 (− 0.18)
Is a security	− 0.819^**^	− 1.139^**^	− 0.941^**^	− 0.938^*^	− 1.003^**^	− 1.291
	(− 2.05)	(− 2.32)	(− 2.13)	(− 1.75)	(− 2.12)	(− 1.61)
	(− 1.98)	(− 1.92)	(− 1.58)	(− 1.18)	(− 1.83)	(− 1.17)
Team lockup period	0.287^*^ (1.91)	0.278^*^ (1.81)	0.268 (1.50)	0.238 (1.02)	0.208 (0.89)	0.481
Product or prototype	0.343 (1.39)	0.248 (0.78)	0.249 (0.91)	0.218 (0.56)	0.307 (1.20)	0.237 (0.55)
High-quality advisory team	0.346 (1.59)	0.136 (0.40)	0.237 (0.98)	0.021 (0.06)	0.309 (1.23)	− 0.072 (− 0.18)
KYC/AML procedure	0.641^***^ (2.73)	0.557^*^ (1.93)	0.634^***^ (2.82)	0.680^**^ (2.29)	0.567^**^ (2.64)	0.642^*^ (1.79)
Has VC backing	0.169 (0.50)	0.343 (0.84)	0.176 (0.48)	0.261 (0.56)	0.100 (0.23)	0.374 (0.49)
Ln(Hard cap size) (USDm)	0.745^***^ (4.69)	0.744^**^ (2.32)	0.832^***^ (4.04)	0.872^**^ (2.53)	0.737^***^ (3.24)	0.975^*^ (1.77)
Month-year FE	Yes	Yes	Yes	Yes	Yes	Yes
Industry FE	No	No	Yes	Yes	Yes	Yes
Country-FE	No	No	No	No	Yes	Yes
*N*	92	57	92	57	92	57
*R*^2^	0.57	0.55	0.60	0.63	0.69	0.79

The table shows regression results of an ordinary least squares regression of the number of ICO contributors on ICO characteristics. We exclude ICOs for which we cannot identify with certainty from which Ethereum address the tokens have been initially distributed. Furthermore, transfers where the amount transferred is worth less than 50 USD or where the receiving address holds more than 10% of the total token supply are excluded. All variables are defined in “Appendix 2.” Columns 3 and 4 include seven industry fixed effects for the following industries: Blockchain Infrastructure, Entertainment, Finance, Payments, Trading and Exchanges, Business Services and Other Software. For the estimation of country fixed effects in columns 5 and 6, countries with less than five observations have been grouped into the categories “Other European,” “Other Asian,” “Other Americas” and “Other Rest of World.” Dependent and independent variables have been winsorized at the 1 and 99% level. *T*-statistics calculated from robust standard errors are listed in parentheses below the coefficients. One, two and three asterisks indicate statistical significance at the 10, 5 and 1% level, respectively

Many of the characteristics related to investor protection are statistically significant at the 10–5% level. Tokens that are unambiguously securities, i.e., grant their holders cash flow rights, surprisingly get 68.0% fewer investors. One possible explanation for this fact might be that such tokens are associated with more legal uncertainty. A one standard deviation increase in the founder lockup period on the other hand increases the number of investors by 29.5%, whereas a one standard deviation increase in the fraction of tokens retained by the founders is associated with a 46.5% decrease in the number of investors. A likely explanation is that a large founder share increases the risk of dilution for the investors in case the founders decide to sell the tokens in the secondary market.^20^ Columns 3 and 4 include seven industry fixed effects for the following industries: Blockchain Infrastructure, Entertainment, Finance, Payments, Trading and Exchanges, Business Services and Other Software. The results on the main independent variables are quantitatively and qualitatively similar to the results reported in columns 1 and 2. In columns 5 and 6, we add in addition country fixed effects to the regression. Most of the results are comparable with the other results without country fixed effects, with the exception of the indicator variable for the ICO having a presale that loses its statistical significance in column 5.

In unreported regressions, we have also included the number of white paper pagers as an additional explanatory variable. The length of the white paper does not have explanatory power in any of the regressions.

Overall, the alignment of incentives between founders and investors seems to matter more for contributors’ investment decision than the level and quality of disclosure. Finally, ex ante larger ICOs attract more investors as every 1% increase in the hard cap is associated with a 0.7% increase in the number of investors.

### Fraction of repeat contributors

Perhaps surprisingly, the vast majority of addresses in our investor sample only contributes to one ICO. Only 19.1% of addresses contribute more than once, and only 1.1% of addresses participate at least five times.^21^ To address the possible concern that our limited sample of ICOs is the reason for this result, we also analyze a comprehensive sample of primary and secondary market token purchases using a sample of all ERC20 tokens listed on coinmarketcap.^22^ The results for this extended sample are very similar and indicate that only 19.6% of investors hold more than one type of token over the sample period, and only 1.3% hold five or more.

There is evidence suggesting that more professional investors contribute more frequently, however. Table 6 displays the results from regressing the average size of the contributions made by an Ethereum address on the number of ICOs in which the address participates over the sample period. The logarithmic specification in column 1 implies that the average contribution increases by 27.1% when the number of ICOs the address has contributed to increases by one, statistically significant at the 1% level. Column 2 presents a linear specification. The coefficient estimates indicate that the mean investment made by an address increases by 264.6$ when the total number of ICOs invested in is increased by one, also significant at the 1% level.

**Table 6:** Number of contributions by address and mean investment amount

	Ln(mean investment in USD)	Mean investment in USD
	(1)	(2)
Number of ICOs invested in	0.240*** (89.61)	264.598*** (19.74)
Constant	6.108*** (1241.49)	1830.200*** (81.07)
*N*	257,073	257,073
*R*^2^	0.02	0.00

The unit of observation is an Ethereum address that has contributed to at least one of the ICOs in the investor sample. Coefficients are estimated using ordinary least squares. Dependent and independent variables have been winsorized at the 1 and 99% level. *T*-statistics calculated from robust standard errors are listed in parentheses below the coefficients. One, two and three asterisks indicate statistical significance at the 10, 5 and 1% level, respectively

Studying the portfolio allocation decisions of individual investors in the stock market, Goetzmann and Kumar (2008) find that the average investor holds only four stocks in his account at a large online brokerage firm. Our sample investors use the same address to invest in 1.3 different tokens through the primary market on average. If we count Ether as a separate financial asset and add secondary market purchases, the average tokenholder invests into 2.4 different assets on the Ethereum blockchain over the sample period. It is likely that ICO investors also hold cryptocurrencies on other blockchains such as Bitcoin that we cannot link to their Ethereum wallet. So, the number of cryptoassets the average investor holds appears to resemble the number of assets that individual investors have been found to own in the stock market. A caveat regarding this conclusion is that ICO investors might be using different wallets for different ICOs, which we cannot rule out completely (similar to the concern that clients of the online brokerage studied by Goetzmann and Kumar (2008) may have multiple security accounts at different banks). But given the evidence presented in Sect. 4.1 and the fact that repeat contributors invest larger amounts, we deem it possible but unlikely.

### What motivates investors to participate in ICOs?

#### Are contributors motivated primarily by financial returns?

Ex ante, we see two primary reasons why people might participate in ICOs. The first is to make a financial profit and the second to prepurchase the product or service the issuer is developing.

The majority of our sample tokens are either utility tokens or security tokens. For the 22% of tokens that we classify as security tokens because they pay dividends, interest or make other financial distributions, the nature of the token makes it likely that investors are motivated primarily by financial gains. For utility tokens, which represent 61% of our sample, the answer requires more investigation.

To determine what motivates investors to buy utility tokens, we study the frequency of trading in secondary markets, which we see as an indication of investors having a financial motivation rather than mainly prepurchasing a product. We calculate the fraction of ICO investors that sell at least one token within 90 days of the ICO, as well as the fraction of the ICO allocation resold by investors over the same time period. We explicitly restrict our sample to platforms that are in the prelaunch phase by manually collecting platform launch dates and dropping observations for tokens in the post-launch period.

While token transfers from one address to another are publicly visible on the Ethereum blockchain, it is more difficult to infer the purpose of such transfers from the data. There are three main reasons for token transfers: investors spend tokens to consume the product, investors move tokens to a different wallet, or investors sell tokens in the secondary market. We exclude the first reason by restricting our sample to token transfers that occur before the launch of the service or product. We have shown in Sect. 4.1 that daily token transfers correlate very highly with exchange trading volume of the same token, so token transfers between wallets belonging to the same investor do not make up a significant fraction of token transfer either. Hence, the main purpose for token transfers in this sample is sales of tokens on exchanges.

**Fig. 2:**
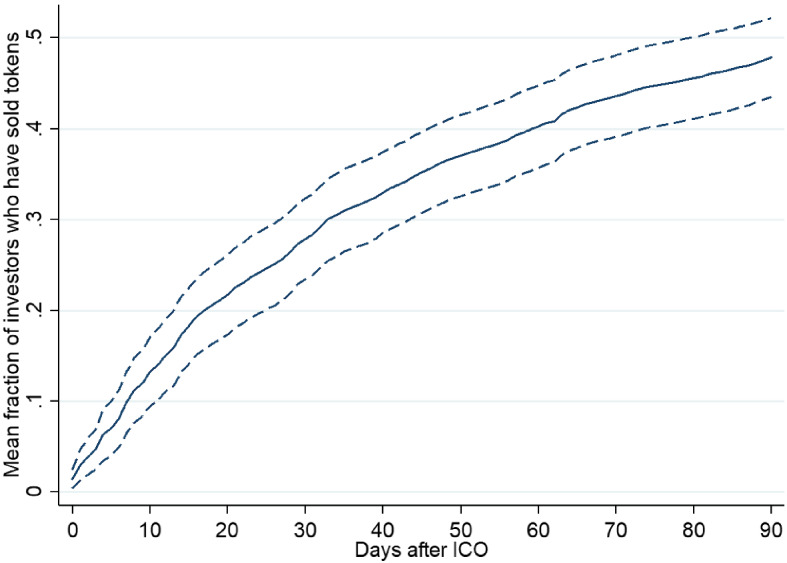
Token sales within 90 days of the ICO. The figure displays the mean fraction of participants per ICO that have sold at least one token within 90 days following the close of the crowdsale. The sample consists of 98 ICOs conducted on the Ethereum platform but is limited to the prelaunch phase during which investors cannot yet use utility tokens for the designated product. Dashed lines indicate the 95% confidence interval for the mean

We find that a substantial fraction of investors sell their allocation soon after the ICO. We estimate that, for utility tokens, on average 49.3% of all investors sell some or all of their tokens within 90 days of the ICO.^23^ Over the same time window, the mean number of tokens, net of new purchases, sold by the original ICO investors in the secondary market following the crowdsale, scaled by the total number of tokens distributed in the ICO, amount to 41.8%. Therefore, a substantial fraction of contributors who purchase utility tokens sell a sizeable portion of their holdings before the product is developed and usable. We observe similar behavior in the full sample of tokens, which includes securities and cryptocurrencies. Figure 2 graphs the mean (across ICOs) fraction of investors who have sold tokens over time. The fraction is monotonously increasing over time and grows particularly rapidly over the first 3 weeks. After the first week, 8.1% of investors have sold at least one token. After 2 weeks, the fraction reaches 15.8%. After 1 and 2 months, the fraction of sellers is 27.3% and 39.9%, respectively. After 90 days. 47.9% of investors have sold at least one token. Net token sales by ICO contributors over the same time window amount to 42.3% of the total ICO allocation.

Our results are consistent with the survey evidence provided by Fisch et al. (2018). Out of a sample of 517 ICO investors, 50.7% of participants answered that a “future sale of the token at a higher price (shortly after the ICO)” was an “important” or “very important” reason for their investment decision.

#### What properties of ICOs make them attractive to investors?

Having established that ICO participants are often small investors motivated by the prospect of financial returns, a natural follow-up question to ask is what features might make ICOs so attractive to retail investors. One potential explanation is that they have “lottery features”: high idiosyncratic volatility, high skewness and a low absolute price. Individual investors in stock markets have been shown to have a preference for stocks with such characteristics (e.g., Kumar 2009). In our sample of ICOs, the average annualized volatility of returns in excess of Ethereum for the 9 months following the ICO is 173%. Returns are also positively skewed (5.13) in the cross section, and the median token has a price of only $0.16 during the crowdsale.

In addition, researchers have shown that investors fear to miss out in new industries with large growth potential and uncertainty and do not necessarily carry out the required due diligence. A substantial body of evidence comes from the last period of technological revolution, the internet boom. Cooper et al. (2001) examine 95 firms that changed their names to a “.com” firm. These small firms, mostly traded on the OTC Bulletin Board, experienced 53% 5-day announcement returns on the news of the name change. Lamont and Thaler (2003) show that investors valued carve-outs of technology stocks irrationally high during the same boom period. Griffin et al. (2011) show that during the technology stock reversal in March 2000, institutional investors sold technology stocks to retail investors (especially those without financial advisors). It seems that retail investors in ICOs could be driven by the same motivation that drove retail investors during the internet boom.

Shiller (2000) uses the term “new era economic thinking” to describe the tendency of technological innovation to lead to financial expansions. In Shiller’s words, “stock market expansions have often been associated with popular perceptions that the future is brighter or less uncertain than it was in the past.” This thinking is often linked to the emergence of new technologies, as “the public is interested in expansive descriptions of future technology—for example, in what amazing new capabilities computers will soon have—not in gauging the level of US corporate earnings in coming years.” The emergence of blockchain technology and its potential to disrupt the financial system presents a potential trigger for such new era thinking, which could provide an additional explanation for the large number of retail investors participating in ICOs.^24^

#### Do the large discounts to presale investors impact their trading behavior?

There is substantial heterogeneity among ICO investors. Some invest larger amounts and do so more frequently and may behave in a different way. Presale investors in particular usually invest more, receive a significant discount over crowdsale investors and can thus lock in a profit by selling their allocation in the secondary market directly after the ICO. This situation is akin to flipping IPO share allocations on the first trading day to benefit from underpricing (e.g., Aggarwal 2003 or Krigman et al. 1999). As long as the secondary market price lies at or above the presale price, and the market is sufficiently deep, investing in the presale could thereby be profitable regardless of the issuer’s fundamentals. We therefore expect presale investors to have a particularly short time-horizon.

If it is common for presale investors to “flip” their investment in this manner, the correlation between the size of the investment and the holding period should be negative.^25^ We estimate a regression of the number of days until the first sale of tokens by an investor, measured from the last day of the ICO, on the amount contributed. At the time of the analysis, we have 9 months of post-ICO data for the last sample ICO; therefore, the dependent variable for this test is right-censored at 270 days.

Table 7 displays results from Tobit regressions where both the dependent and independent variables are in natural logarithms. The specifications in columns 1 and 2 suggest that there is a negative relationship between the size of the contribution and the holding period, statistically significant at the 1% level, implying that larger investors sell earlier. The specification in column 1 implies that a 1% increase in the contribution decreases the (latent, uncensored) holding period by 0.5%. The specification in column 2 adds ICO fixed effects that control for observable and unobservable ICO-level characteristics.^26^ The estimate from the fixed effects specification suggests that a 1% increase in the investment amount decreases the holding period by 0.2% on average. In column 3, we included the contribution amount squared to examine potential nonlinear effects. We find that indeed, the largest investors tend to hold the tokens longer. In column 4, we interact the size of the contribution with an indicator variable equal to one if the ICO had a presale, and zero otherwise. The interaction term is negative and statistically significant at the 1% level, suggesting that the relationship between size and holding period is stronger in ICOs that have a presale. The coefficient for the interaction term amounts to about a third of the magnitude of the relationship between the size and holding period estimated in column 2. While the coefficient on the contribution amount by itself decreases by around 20% in column 3, it retains its statistical significance, suggesting that larger investors still sell earlier in ICOs that do not have a presale. A partial explanation for the negative correlation in those ICOs might be that some issuers grant volume-based discounts to crowdsale investors. Column 5 provides an additional specification in which we interact the size of the contribution with the presale discount, based on the subsample of ICOs that had presale. The coefficient estimate for the interaction term is negative and statistically significant at the 1 % level, implying that large investors sell earlier if the presale discount was larger, i.e., when it is more likely that the secondary market price after the initiation of trading lies above the presale price. The impact of the presale discount is meaningful in economic terms as well; the marginal effect of the contribution size on the holding period is roughly 14% larger for an ICO with a presale discount at the mean compared to the marginal effect for an ICO with a presale discount of zero. Column 6 shows that this result is robust to controlling for the nonlinear effect of the contribution amount.

**Table 7:** Token holding period as a function of the investment amount

	(1)	(2)	(3)	(4)	(5)	(6)
Ln(contribution in USD)	− 0.454*** (− 153.87)	− 0.240*** (− 88.33)	− 0.449^***^ (− 24.30)	− 0.189*** (− 43.74)	− 0.257*** (− 32.98)	− 0.499^***^ (− 17.63)
Ln(contribution in USD)^2^			0.015^***^ (11.44)			0.017^***^ (8.90)
Ln(contribution in USD) * presale				− 0.084*** (− 15.08)		
Ln(contribution in USD) * Presale discount					− 0.103*** (− 4.08)	− 0.064^**^ (− 2.52)
Constant	7.672*** (384.48)	6.432*** (73.28)	7.107^***^ (67.17)	6.661*** (74.72)	6.776*** (68.40)	7.519^***^ (58.01)
Token FE	No	Yes	Yes	Yes	Yes	Yes
*N*	264,439	264,439	264,439	264,439	158,575	158,575
Pseudo *R*^2^	0.03	0.12	0.12	0.12	0.09	0.09

The table presents results of Tobit regressions of the number of days until the first sale of tokens by an ICO contributor on the size of the contributor’s investment in US dollars, both in natural logarithms. The unit of observation is an investor in an ICO. The sample used for this test is the “investor sample” consisting of 98 ICOs conducted on the Ethereum platform. The number of days is measured from the last day of the crowdsale period and is right-censored at 270. All continuous variables have been winsorized at the 1 and 99% level. *Presale* is an indicator variable equal to one if the ICO had a presale, and zero otherwise. The *presale discount* is defined as the difference between the maximum crowdsale price and the minimum presale price, measured as a fraction of the former. Standard errors are clustered by ICO. *T*-statistics are reported in parentheses below the coefficient. One, two and three asterisks indicate statistical significance at the 10, 5 and 1% level, respectively

Overall, Table 7 provides evidence that some large presale investors tend to flip their allocations to realize the windfall profits generated by their discount. They display a behavior that is similar to IPO investors that flip their IPO allocations during the first trading days to benefit from IPO underpricing (e.g., Aggarwal 2003). Our analysis has important consequences for crowdsale investors who rely on presale investors for ICO certification. Unlike the investments of early-stage investors in typical seed rounds that are illiquid, presale investors can obtain liquidity on the secondary market. The value of their certification may be less than crowdsale investors believe, especially when presale investors obtain large discounts.

## Investor protection

As illustrated by the extended summary statistics in “Appendix 3,” our average sample firm was founded only 1.6 years prior to the ICO, has 11 employees and does not have a finished product. Hence, it is at a stage in its life cycle when it would typically seek angel or venture capital funding instead of going to public markets.

Asymmetric information and moral hazard problems between entrepreneurs and financiers are a prominent issue in early-stage financing. Therefore, investment contracts between venture capitalists or angel investors and entrepreneurs usually provide numerous protections to investors, such as cash flow rights, board and voting rights and liquidation rights (Kaplan and Strömberg 2003). Our goal in this section is to determine whether the retail investors who participate in ICOs receive some of the protections that professional investors typically ask for.

### Cash flow rights

Residual cash flow rights in ICOs are rare and are only present among a subset of the 22% of ICOs that issue security tokens. For the vast majority of ICOs, investors will only receive financial gains from their token holdings if the product developed by the issuer gains in popularity. In addition to the lack of dividends, there is also a more subtle point with selling utility tokens. Whether and how much the price of a utility token increases with the popularity of the product depends on the issuer not accepting alternative means of payments in the future (e.g., Catalini and Gans 2018). Accepting other means of payment decreases the demand for tokens sold in the ICO and subsequently decreases the value of the token. Interestingly, token sales terms rarely expressively prohibit the issuer from introducing additional means of payments.

### Liquidation preferences

Liquidation preferences are an important element of term sheets between venture capitalists and entrepreneurs, most commonly in the form of convertible preferred stock. Liquidation preferences reduce moral hazard concerns: Should the company fail, merge or be sold, VC investors receive the first proceeds, typically up to their initial investment. Kaplan and Strömberg (2003) study a sample of VC financing rounds and find that over 96% use preferred stock. Token sales agreements on the other hand typically state that the firm will make a “best effort” attempt to deliver the promised product, but investors have no additional rights in case of failure and liquidation.

### Voting rights, board of directors and staggered distribution of ICO proceeds

Investors only have voting rights in 18% of ICOs, and votes are usually non-binding in nature and limited to approving major investment decisions or updates of software protocols. We are not aware of any firm that allows ICO investors to participate in director elections. VCs, on the other hand, control 41.4% of board seats and a majority of the shareholder votes following the average financing round (Kaplan and Strömberg 2003). According to the same source, 14.6% of venture funding rounds place restrictions on the release of committed funds. In contrast, only 4% of ICOs specify milestones for the release of funds, and only 4% leave an independent custodian in charge of the funds raised by the company.

### Lockup periods

Firms lock up at least part of the tokens held by them and their founders in a majority (59%) of ICOs, compared to 41% of VC contracts containing vesting clauses for founders (Kaplan and Strömberg 2003). The mean weighted average lockup period of the tokens retained by the issuing firm and its founders is 1.1 years.

Another concern for investors should be that presale investors, who usually purchase tokens at a substantial discount, could realize a profit by selling the tokens directly following the ICO in the secondary market, which would put downward pressure on prices. Investors in initial public offerings are exposed to a similar risk, because of early investors and insiders who typically own a large share of the company going public and might be looking to sell soon after the IPO. For this reason, most IPOs feature a lockup period that typically lasts for 180 days during which pre-IPO shareholders are barred from selling (Field and Hanka 2001; Brav and Gompers 2003). ICOs rarely address this concern, although investors would probably benefit given our finding that presale investors often quickly sell their allocation in secondary markets after the ICO is over. Only 14% of ICOs impose a lockup period on presale investors. For those that do, presold tokens remain locked up for 0.53 years on average following the ICO.

### Control rights in angel investments

ICOs fund projects in the early stages of product development. Contractual protections of angel investors are therefore perhaps a better benchmark than protections of venture capitalists. Goldfarb et al. (2014) and Wong et al. (2009) examine the contractual provisions that angel investors request and compare them with the provisions of venture capitalists. They generally find that the angel market is more informal than the venture capital market and has fewer control rights. However, both papers demonstrate that angel investors do receive control rights. For example, Goldfarb et al. (2014) show that in their sample, most angels get preferred stock with liquidation preferences. Wong et al. (2009) show that in their sample, angel investors get board seats in slightly less than 50% of deals and that they take straight equity without liquidation preferences in about one-third of deals.

Angels make up for the lack of more detailed control rights by geographical proximity and deep industry experience. It is unlikely that ICO investors have the same geographical proximity; ICOs are typically marketed globally and the whitepaper (a document that illustrates the product, the team and the ICO in broad strokes) provided by the issuer is often translated into multiple languages. We do not know the level of industry experience of the typical ICO investor, but speculate that it is lower than for the typical angel investor, given the low contribution amount and large number of contributors in ICOs relative to angel investments.

## Empirical analysis of ICO secondary market returns

We now examine how ICO investors fared in secondary markets. Did investors obtain a positive return on their ICO investments, despite the risks inherent in investing in ICOs and the lack of investor protection? Do measures that could reduce information asymmetries and substitute for the oversight typically provided by financial intermediaries have explanatory power for ICO returns and could they serve as a guideline for investors to choose ICOs? Do contributor characteristics such as number of contributors or average contribution size help predict returns?

### Return summary statistics

**Fig. 3:**
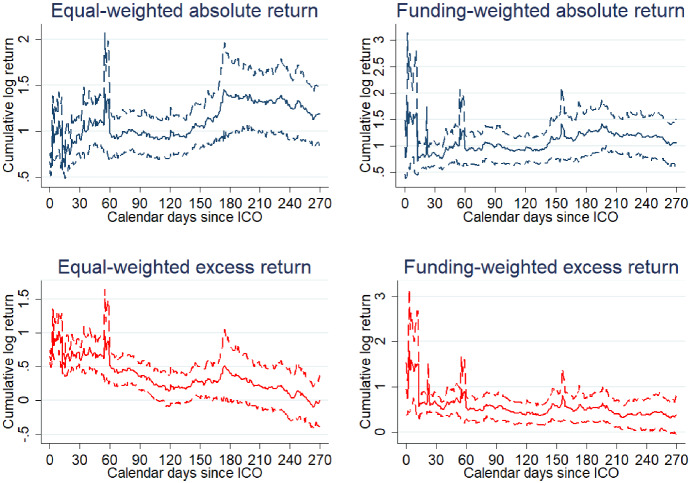
Secondary market performance of all ICOs. The figure is based on the secondary market prices for 250 ICOs. Returns are continuously compounded price returns based on the average price paid by investors in the crowdsale. If the average crowdsale price is unavailable, returns are based on the mid-price (average between highest and lowest price paid in the crowdsale). Observations with daily trading volume below $1000 have been excluded. Funding-weighted returns have been weighted by total funding received during the crowdsale. Excess returns are in excess of the return on the Ethereum cryptocurrency. Dashed lines indicate the 95% confidence interval for the mean; confidence intervals have been bootstrapped using 250 replications

Figure 3 displays four graphs of the secondary market performance of all sample ICOs. The left-hand side of the figure shows equal-weighted returns, and the right-hand side funding-weighted returns. The top two graphs show absolute returns, and the bottom two graphs show returns in excess of the return on Ether. We choose a period of 270 days (9 months) post-ICO, because it is the longest period that is complete for all sample ICOs as of the time of writing. Secondary market and crowdsale prices are available for 250 out of 306 ICOs. We exclude thinly traded observations with daily trading volume below $1000. Furthermore, we use the last observed cumulative return for the remainder of the sample period in case a token is delisted. Delistings happen for twelve sample ICOs. If price data for a token are missing intermittently, we treat the cumulative return for the period without price data as missing as well.

**Fig. 4:**
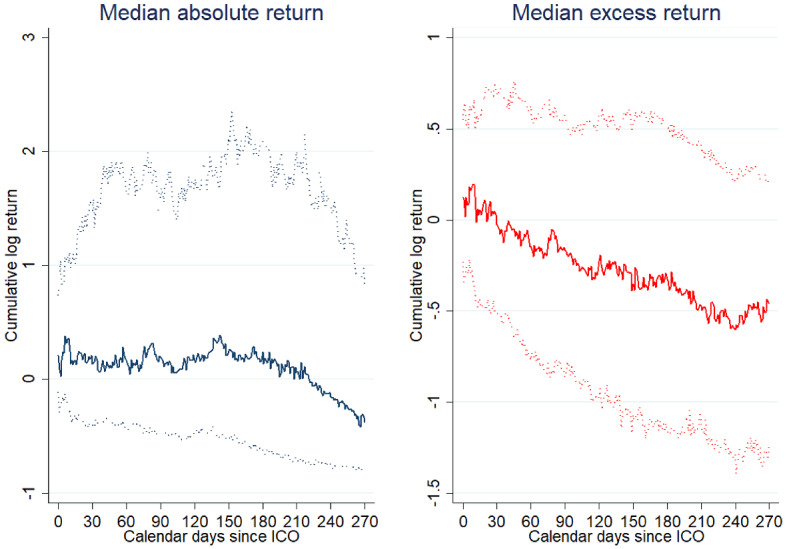
Median secondary market performance. The figure is based on the secondary market prices for 250 ICOs. Returns are continuously compounded price returns based on the average price paid by investors in the crowdsale. Where the average crowdsale price is unavailable, returns are based on the mid-price (average between highest and lowest price paid in the crowdsale). Observations with daily trading volume below $1000 have been excluded. Solid lines represent the median absolute return and the return in excess of the Ethereum cryptocurrency, respectively. Dotted lines indicate the 25th and 75th percentile

Crowdsale investors gain on average 118.7% over a period of 270 days following the ICO. The figure further displays a weighted average return based on the total amount of funding raised during the ICO’s crowdsale stage. The results indicate a 105.7% return over 9 months.^27^ We isolate the performance of individual ICOs from that of the market for cryptocurrencies in general by calculating returns in excess of the Ethereum cryptocurrency (results are comparable when we use the return on Bitcoin for reference instead). Excess returns amount to 0.7% for the full sample using equal weights and 38.4% using value weights. The results suggest that the underlying value of Ether drives much of the returns of ICO investors. Furthermore, the distribution of ICO returns is positively skewed. Figure 4 displays medians for absolute and excess returns. Both are negative for the median ICO after 270 days, implying that a minority of ICOs is driving the positive average returns shown in Fig. 3. Our result emphasizes the lottery-like features of ICOs.

Overall, our estimates are more conservative than those of existing research on the market performance of ICOs, in particular Dittmar and Wu (2019) and Benedetti and Kostovetsky (2018). Dittmar and Wu find raw returns of 362.21% and Bitcoin-adjusted returns of 92.08% over a window of 180 calendar days for 570 ICOs. Benedetti and Kostovetsky find raw returns of 430.9% and Bitcoin-adjusted returns of 242.5% over the same window for a sample of 293 ICOs. It is possible that our sample of large successful ICOs is less prone to price manipulation or microstructure effects, which would explain the different findings.

Given the overall lack of disclosure and investor protection and the large number of likely uninformed retail investors, it is surprising that returns for the average ICO are positive after 9 months. Lamont and Thaler (2003) argue that both frictions such as short sales constraints and irrational investors were needed for mispricing of technology stocks to persist during the tech bubble. They show that it was very difficult to short the overpriced carved out technology stocks of their sample so that the arbitrage opportunity could persist. Ofek and Richardson (2003) use a model with short sale restrictions to explain the internet bubble. Interestingly, Cheng et al. (2019) show that the positive short-term announcement returns to the usage of blockchain technology eventually reverse for publicly listed firms that do not have any expertise in the technology and for which short sales are much easier than for ICO tokens.

At this point in time, we cannot assert with certainty if the value of tokens is justified, perhaps due to the technological advances of the platform and products offered, or whether token valuation is a speculative bubble that may deflate in the future.

### Determinants of returns

Table 8 presents regression results of the (continuously compounded) financial return to crowdsale investors 270 days following the ICO on investor and ICO characteristics. Returns are based on the average crowdsale price or the mid-price (average between the maximum and minimum crowdsale price) where the average is not known. In addition, all specifications control for the return on the Ethereum and Bitcoin cryptocurrencies over the same 270 days to isolate the performance of the individual ICO from overall market trends. We also add time fixed effects for the month of the last day of the ICO, when trading in secondary markets typically starts. We acknowledge that absent a risk model, we are unable to distinguish initial mispricing (either by the issuer, or where an auction mechanism is used, by investors) from compensation for risk in the secondary market regressions.

**Table 8:** Determinants of return 270 days after the ICO

	Fundraising		Business	Governance	All characteristics		Investor base	
	(1)	(2)	(3)	(4)	(5)	(6)	(7)	(8)
Has VC backing	0.501^**^ (2.29)	0.423^*^ (1.79)			0.319 (1.39)	0.365 (1.37)		0.712^**^ (2.47)
Unsold tokens burnt	− 0.019 (− 0.09)	− 0.000 (− 0.00)			− 0.020 (− 0.09)	− 0.066 (− 0.26)		0.555^**^ (2.15)
Ln(1 + presale amount raised)	− 0.130 (− 1.52)	− 0.259^*^ (− 1.90)			− 0.183^*^ (− 1.92)	− 0.406^**^ (− 2.62)		0.091 (0.66)
Ethereum return	1.244^**^ (2.56)	0.533 (0.84)	0.804^**^ (2.04)	0.913^**^ (2.28)	1.119^**^ (2.16)	0.647 (0.85)		0.313 (0.42)
Bitcoin return	− 0.721 (− 0.92)	0.155 (0.15)	0.260 (0.39)	0.234 (0.34)	− 0.467 (− 0.59)	0.005 (0.00)		1.711^*^ (1.74)
Presale discount		− 1.333^**^ (− 2.19)				− 1.238^*^ (− 1.81)		
Presale lockup period		0.373 (0.35)				− 0.461 (− 0.48)		
Product or prototype			0.015 (0.09)		− 0.151 (− 0.74)	− 0.123 (− 0.45)		0.527^**^ (2.23)
Experienced team			0.183 (1.04)		0.063 (0.29)	0.095 (0.34)		0.680^*^ (1.97)
High-quality advisory team			0.150 (0.86)		0.280 (1.33)	0.353 (1.37)		0.252 (0.78)
Project code available			0.532^***^ (3.14)		0.467^**^ (2.15)	0.312 (0.94)		0.739^**^ (2.49)
Use of proceeds mentioned			− 0.067 (− 0.33)		0.063 (0.21)	0.060 (0.18)		− 0.048 (− 0.17)
Offshore incorporation				− 0.149 (− 0.62)	0.117 (0.39)	0.018 (0.05)		0.620^*^ (1.78)
Legal form and jurisdiction known				0.314 (0.90)	0.365 (0.89)	0.035 (0.08)		− 0.401 (− 0.62)
KYC/AML procedure				0.245 (1.18)	0.121 (0.49)	0.248 (0.85)		0.564^*^ (1.92)
Token share team (ex ante)				0.100 (0.20)	− 0.136 (− 0.22)	− 0.388 (− 0.47)		− 1.542^*^ (− 1.91)
Team lockup period				0.175^*^ (1.96)	0.204 (1.62)	0.501^***^ (3.27)		0.109 (0.66)
Legal advisor disclosed				− 0.013 (− 0.07)	0.061 (0.28)	0.278 (0.98)		0.002 (0.01)
Ln(number of contributors)							0.113 (0.82)	0.003 (0.03)
Ln(median contribution size)							− 0.104 (− 0.53)	− 0.365 (− 1.64)
Month FE	Yes	Yes	Yes	Yes	Yes	Yes	Yes	Yes
Industry FE	Yes	Yes	Yes	Yes	Yes	Yes	Yes	Yes
*N*	207	108	261	251	199	106	71	70
*R*^2^	0.61	0.61	0.60	0.59	0.64	0.66	0.72	0.89

The table shows OLS regressions of ICO returns on ICO characteristics. The dependent variable is the log return based on the average crowdsale price 270 calendar days following the completion of the ICO. If the average price is unavailable, the return is calculated based on the mid-price (average between the maximum and minimum crowdsale price). If an ICO is delisted before 270 days of trading, the return is based on the last price before delisting. All variables are defined in “Appendix 2.” All regressions include seven industry fixed effects. All continuous variables are winsorized at the 1 and 99% level, respectively. One, two and three asterisks indicate statistical significance at the 10, 5 and 1% level, respectively

The coefficients in columns 4 to 6 indicate that a one standard deviation increase in the lockup period increases holding period returns by 21.4–61.6% points, with statistical significance at the 5% level or below in columns 4 and 6. A possible explanation for this result is that a longer lockup period improves the alignment of incentives between investors and the team. Additionally, columns 3 and 5 indicate that ICOs which disclose the project’s source code ex ante produce 46.7–73.9% points higher holding period returns. Obvious explanations based on mispricing are that the disclosure makes it more likely that the firm will be able to deliver a viable product, or less likely that the ICO is a scam.

Column 6 indicates that holding period returns are negatively correlated with the presale amount, statistically significant at the 5% level. The corresponding estimates in columns 2 and 5 are statistically significant at the 10% level. The coefficient estimates are economically meaningful as well, implying that a 1% increase in the amount raised in the presale leads to a 0.2–0.4% point decrease in the holding period return. An explanation for this result based on mispricing could be that because presale investors receive a discount, they can lock in a profit by selling their tokens in the secondary market directly after the ICO, thereby putting downward pressure on prices. Consistent with this explanation, the coefficient estimates for the presale discount are negative, with statistical significance at the 5% level in column 2. A one standard deviation increase in the presale discount is associated with a 30.9% decrease in the holding period return for crowdsale investors.

Overall, however, the holding period return of the Ethereum cryptocurrency has the largest explanatory power for 9-month ICO returns, both in terms of statistical and economic significance. The corresponding coefficient estimates are statistically significant at the 5% level in columns 1, 3, 4 and 5 and imply that a 1% point increase in the return on Ethereum is associated with a 0.8–1.2% point increase in the holding period return of an ICO.

There is reason to believe that the holding period return might depend on the composition of the investor base. The results presented in Sect. 4 show that larger investors sell their tokens sooner, at least partially due to presale investors who can lock in a profit by selling their tokens right after the ICO, and the tests in this section have established a negative correlation between secondary market returns and the amount of funding raised in the presale. Column 7 therefore presents the results of additional specifications regressing the 9-month holding period return on the number of contributors and the size of the median contribution, both in natural logarithms. Neither variable is statistically significant. When we add the full set of ICO-level controls to the regression in column 8, the coefficient for the median contribution size is negative but its *t*-statistic remains just below the threshold for statistical significance.^28^

## Conclusion

Initial coin offerings are a novel fundraising mechanism for start-up companies, in particular those focusing on applications of the blockchain technology. Our paper characterizes the typical ICO investor and seeks to understand his primary motives to participate in the ICO market.

Based on an analysis of ICOs hosted on the Ethereum platform, we conclude that most contributors are likely to be retail investors. The average ICO has almost 4700 contributors. The median contributor invests a relatively small amount. The ICO market appears to have successfully given access to the financing of innovation to a new class of investors, which is a long-standing public policy issue (e.g., the Jumpstart Our Business Startups Act, or JOBS Act, passed in 2012 in the US, also wishes to encourage the financing of startups by smaller investors).

For at least half of all primary market investors, the goal of participating in the ICO appears not to be the prepurchase of a product that they intend to use but rather speculation, as they sell the tokens before the product is developed. Large presale investors who certify ICOs and whose participation is monitored and relied upon by crowdsale investors (e.g., Howell et al. 2019; Fisch 2019) are potentially conflicted. They buy tokens at a significant discount of 34% average and can lock in a profit by selling their allocation in the secondary market right after the ICO. We show that large investors indeed sell quickly after the ICO, and we find that holding period returns for crowdsale investors are significantly lower in ICOs with a large presale and/or a large presale discount.

ICO returns have features akin to lottery stocks, and most projects feature a new technology that has the potential to lead to dramatic efficiency improvements and new applications. Both of these characteristics have been shown to be of interest to retail investors (e.g., Kumar 2009 or Cooper et al. 2001). These characteristics could explain why retail investors purchased ICOs despite the lack of detailed information on the funded projects and why ICO returns are on average positive 9 months after the ICO. Because blockchain technology is a recent development that has not yielded many economically viable applications, it seems impossible to assert with certainty whether the returns we find are justified, or whether ICO tokens are currently experiencing a speculative bubble that may deflate in the future.

## Appendix 1: Sample Constituents

See Table 9.

**Table 9:** Sample constituents

Name	Date ended	Total raised	Industry	Inv sample	Name	Date ended	Total raised	Industry	Inv. sample
Filecoin	9.7.17	233.0	Data storage	No	Basic Attention Token	5.31.17	36.0	Commerce and advertising	No
Tezos	7.14.17	219.7	Blockchain Infra.	No	hero token	2.28.18	35.9	Finance	No
Hdac	12.22.17	210.2	Blockchain Infra.	No	Centra	10.2.17	35.6	Payments	No
Bancor	6.12.17	159.3	Trading and exchanges	Yes	Aeternity	6.9.17	35.0	Blockchain Infra.	Yes
SIRIN LABS	12.25.17	157.9	Communications	Yes	Enjin Coin	10.31.17	34.8	Video games and VR	Yes
Polkadot	10.27.17	144.3	Blockchain Infra.	No	CRYPTO20	11.30.17	34.8	Finance	No
TenX	6.24.17	104.8	Payments	No	TokenPay	12.26.17	34.2	Payments	No
Status	6.21.17	101.0	Blockchain Infra.	Yes	Etherparty	10.29.17	33.6	Blockchain Infra.	Yes
Envion	1.14.18	100.0	Crypto mining	No	SingularityNET	12.22.17	33.3	Data analytics	Yes
Kik	9.26.17	98.5	Blockchain Infra.	Yes	Jibrel Network	12.27.17	33.2	Finance	No
Grid+	11.11.17	74.7	Energy and utilities	Yes	Civic	6.28.17	33.0	Identity and reputation	No
Bankex	12.26.17	74.3	Finance	No	Raiden Network Token	11.1.17	31.9	Payments	No
WAX	11.29.17	68.4	Video games and VR	No	Polybius	7.5.17	31.0	Finance	No
NAGA	12.15.17	65.0	Payments	Yes	Storiqa	1.29.18	30.2	Commerce and advertising	Yes
Kyber Network	9.18.17	58.7	Trading and exchanges	Yes	Stox	8.3.17	30.0	Gambling	Yes
Blockstack	12.1.17	56.8	Privacy and security	No	Blackmoon Crypto	9.13.17	30.0	Finance	No
Storm	12.7.17	56.2	Video games and VR	No	Restart Energy	3.14.18	30.0	Energy and utilities	Yes
Ambrosus	10.20.17	56.1	Provenance and notary	No	DADI	2.28.18	29.0	Cloud computing	No
Neuromation	1.7.18	56.0	Data analytics	Yes	ETHLend	12.27.17	28.7	Finance	No
indaHash	12.20.17	55.7	Social networks	No	Monetha	9.30.17	28.6	Payments	Yes
Crypterium	1.5.18	55.3	Payments	Yes	Electrify.Asia	3.2.18	27.3	Marketplaces	Yes
MobileGo	5.24.17	53.1	Video games and VR	No	Spectre	12.10.17	27.0	Trading and exchanges	No
TraDove	2.28.18	52.0	Social networks	No	OmiseGO	6.27.17	26.8	Payments	No
Pundi X	1.21.18	49.4	Payments	Yes	Monaco	6.18.17	26.5	Payments	Yes
Unikoin Gold	10.22.17	48.3	Gambling	No	Power Ledger	10.6.17	26.4	Energy and utilities	No
SONM	6.17.17	46.7	Blockchain Infra.	Yes	Everex	8.31.17	26.4	Finance	Yes
Enigma	9.12.17	45.0	Marketplaces	No	FunFair	6.23.17	26.0	Gambling	Yes
Bread	12.24.17	44.0	Payments	Yes	Decentraland	8.17.17	26.0	Video games and VR	
Trade Token	1.7.18	42.5	Trading and exchanges	No	BitClave	12.29.17	25.7	Search	Yes
Electroneum	10.19.17	41.5	Blockchain Infra.	No	Neumark	12.17.17	25.7	Finance	No
Finom	12.30.17	41.3	Finance	No	0x	8.16.17	25.5	Trading and exchanges	No
Bloom	1.1.18	40.0	Finance	No	Tierion	7.28.17	25.4	Privacy and security	No
Mobius	1.18.18	39.0	Communications	No	Aragon	5.17.17	25.0	Blockchain Infra.	No
Ripio Credit Network	11.10.17	37.8	Finance	No	Target Coin	8.31.17	24.9	Finance	No
Medicalchain	2.28.18	24.0	Drugs and healthcare	Yes	Waves	5.31.16	15.8	Blockchain Infra.	No
SophiaTX	12.17.17	23.5	Blockchain Infra.	Yes	Qtum	3.21.17	15.4	Blockchain Infra.	No
FinShi	10.6.17	23.0	Finance	No	Maecenas	10.7.17	15.4	Art and music	Yes
Pillar	7.17.17	22.8	Payments	Yes	Santiment Network Token	7.5.17	15.3	Trading and exchanges	No
BitDegree	12.29.17	22.5	Education	Yes	COPYTRACK	2.9.18	15.1	Privacy and security	Yes
BLOCKv	10.25.17	22.1	Blockchain Infra.	No	Ignis	11.4.17	15.0	Blockchain Infra.	No
KICKICO	9.16.17	22.1	Finance	Yes	AirToken	10.7.17	15.0	Finance	No
Simple Token	12.1.17	21.8	Commerce and advertising	No	Adhive	2.28.18	15.0	Commerce and advertising	No
Selfkey	1.14.18	21.7	Identity and reputation	Yes	Dynamic Trading Rights	12.6.17	15.0	Trading and exchanges	No
UTRUST	11.20.17	21.4	Payments	Yes	Red Pulse	10.8.17	14.9	Finance	No
Debitum Network	2.26.18	20.9	Finance	Yes	Cofound.it	6.7.17	14.6	Finance	Yes
Gladius	2.12.18	20.8	Privacy and security	No	Modum	9.22.17	14.6	Drugs and healthcare	Yes
QLINK	1.19.18	20.6	Communications	No	OAX	7.4.17	14.4	Trading and exchanges	Yes
Aventus	9.6.17	20.0	Events and entertainment	Yes	HelloGold	10.5.17	14.3	Finance	No
Dock.io	2.21.18	20.0	Marketplaces	Yes	AdEx	6.30.17	14.2	Commerce and advertising	No
aXpire	1.12.18	20.0	Finance	No	DIMCOIN	8.28.17	14.0	Trading and exchanges	No
Covesting	12.31.17	19.9	Finance	Yes	Substratum	9.14.17	13.8	Blockchain Infra.	No
TE-FOOD	2.22.18	19.1	Provenance and notary	Yes	MicroMoney	11.18.17	13.5	Finance	No
DMarket	11.28.17	19.0	Video games and VR	Yes	DEEX	2.28.18	12.9	Trading and exchanges	No
Uptoken	12.15.17	18.9	Payments	Yes	TokenCard	5.2.17	12.7	Payments	Yes
FintruX Network	2.28.18	18.9	Finance	No	ETCWin	11.6.17	12.4	Blockchain Infra.	No
OriginTrail	1.17.18	18.5	Provenance and notary	Yes	Qbao	11.20.17	12.4	Social networks	No
Lympo	2.28.18	18.2	Health	Yes	Science Blockchain	11.22.17	12.3	Finance	No
Insights Network	2.14.18	18.1	Data analytics	Yes	NapoleonX	2.28.18	12.3	Finance	Yes
Cosmos	4.6.17	18.1	Blockchain Infra.	No	Gnosis	4.22.17	12.1	Data analytics	No
Uquid Coin	11.7.17	17.8	Payments	No	Bitbounce	8.29.17	11.8	Communications	No
Cryptopay	10.30.17	17.5	Payments	No	WaBi	1.28.18	11.8	Provenance and notary	Yes
InsurePal	1.16.18	17.4	Insurance	No	iExec RLC	4.19.17	11.7	Cloud computing	Yes
BOScoin	5.10.17	17.4	Blockchain Infra.	No	REAL	9.30.17	11.2	Real estate	Yes
Mysterium	5.30.17	17.3	Privacy and security	No	Po.et	8.8.17	11.0	Content mgmt.	No
Latium	1.18.18	17.2	Marketplaces	No	Wagerr	6.21.17	10.8	Gambling	No
MCAP	5.27.17	17.1	Finance	No	spectiv	12.29.17	10.7	Commerce and advertising	No
Change	10.16.17	17.0	Finance	Yes	Iconomi	9.26.16	10.7	Finance	Yes
Cindicator	10.12.17	16.9	Finance	Yes	Patientory	6.28.17	10.7	Drugs and healthcare	Yes
Rivetz	9.10.17	16.9	Privacy and security	No	Mercury Protocol	11.24.17	10.5	Communications	Yes
SmartMesh	12.3.17	16.8	Communications	Yes	Devery	1.19.18	10.4	Privacy and security	Yes
AppCoins	12.20.17	16.7	Marketplaces	Yes	ZrCoin	6.9.17	10.4	Commodities	No
Bluzelle	1.20.18	16.3	Data analytics	No	adbank	1.6.18	10.4	Commerce and advertising	No
AidCoin	1.16.18	16.2	Charity	Yes	XPA	8.30.17	10.0	Events and entertainment	No
Arbidex	2.28.18	16.0	Trading and exchanges	Yes	Blockchain Capital	5.10.17	10.0	Finance	No
Gatcoin	1.14.18	16.0	Commerce and advertising	No	doc.ai	10.12.17	10.0	Drugs and healthcare	No
district0x	8.1.17	9.8	Marketplaces	No	DCORP	6.29.17	5.1	Finance	No
Rialto	7.4.17	9.7	Trading and exchanges	Yes	SportyCo	12.10.17	5.1	Finance	Yes
B2BX	11.17.17	9.5	Trading and exchanges	Yes	WeTrust	4.12.17	5.0	Finance	Yes
TIES Network	10.18.17	9.2	Social networks	Yes	Flixxo	11.23.17	4.7	Content mgmt.	Yes
Indorse	9.7.17	9.2	Social networks	No	Sharpe Capital	2.5.18	4.2	Finance	Yes
Starbase	11.24.17	9.0	Finance	No	DECENT	11.6.16	4.2	Content mgmt.	No
BitDice	9.15.17	8.7	Gambling	Yes	Starta	7.5.17	4.1	Finance	No
CarTaxi Token	10.31.17	8.7	Marketplaces	No	Hacken	11.30.17	4.1	Privacy and security	No
EBCoin	2.13.18	8.5	Tourism	No	MiniApps	12.19.17	3.9	Blockchain Infra.	Yes
Inspeer	2.5.18	8.5	Finance	No	Aigang	12.15.17	3.9	Insurance	No
Tomocoin	3.1.18	8.4	Blockchain Infra.	Yes	ALIS	9.29.17	3.8	Social networks	No
Golem	11.13.16	8.3	Cloud computing	Yes	Suretly	8.11.17	3.5	Finance	No
IP Exchange	3.5.18	8.1	Marketplaces	Yes	Lunyr	4.28.17	3.4	Content mgmt.	Yes
Primalbase Token	7.26.17	7.9	Real estate	No	Divi	11.25.17	3.3	Payments	No
GUTS	12.13.17	7.7	Events and entertainment	Yes	Humaniq	4.26.17	3.2	Finance	No
NVO	6.27.17	7.6	Trading and exchanges	No	SRG	1.15.18	3.2	Commerce and advertising	No
iXledger	7.13.17	7.6	Finance	Yes	Proof Suite	12.1.17	3.1	Finance	No
CrowdWiz	1.31.18	7.2	Finance	No	Kibo Lotto	11.9.16	3.1	Gambling	No
Peerplays	5.14.17	7.2	Gambling	No	Mirocana	12.19.17	3.0	Finance	No
TaaS	4.27.17	7.2	Trading and exchanges	No	Ethereum Movie Venture	5.15.17	3.0	Events and entertainment	Yes
Aditus	12.20.17	7.1	Commerce and advertising	Yes	Privatix	11.16.17	2.9	Communications	Yes
BlockCAT	8.18.17	7.0	Payments	No	Melonport	2.15.17	2.9	Finance	No
Blocktix	7.28.17	7.0	Events and entertainment	Yes	SkinCoin	7.21.17	2.8	Video games and VR	Yes
ATLANT	10.31.17	7.0	Real estate	Yes	Lykke	10.10.16	2.8	Trading and exchanges	No
Datum	11.29.17	6.8	Commerce and advertising	Yes	CryptoPing	6.25.17	2.6	Trading and exchanges	No
Hubii Network	9.8.17	6.6	Content mgmt.	No	Genie	2.28.18	2.5	Finance	No
Oxycoin	10.1.17	6.3	Finance	No	Sola	12.25.17	2.2	Social networks	No
Lisk	3.21.16	6.3	Blockchain Infra.	No	Bounty0x	12.16.17	1.9	Search	No
Opus	8.24.17	5.8	Content mgmt.	Yes	vSlice	12.12.16	1.8	Gambling	No
Sociall	9.15.17	5.7	Social networks	No	Wings	1.6.17	1.8	Finance	No
Matchpool	4.4.17	5.6	Social networks	No	SunContract	7.25.17	1.8	Energy and utilities	Yes
DigixDAO	3.30.16	5.5	Finance	No	Blockpool	6.30.17	1.8	Blockchain Infra.	No
FirstBlood	9.26.16	5.5	Video games and VR	No	Smart Investment Fund Token	9.15.17	1.7	Finance	Yes
Synereo	10.18.16	5.4	Social networks	No	FundYourselfNow	7.31.17	1.6	Finance	No
Chronobank	2.14.17	5.4	Marketplaces	Yes	FidentiaX	12.6.17	1.6	Insurance	Yes
TrueFlip	7.27.17	5.4	Gambling	No	Incent	11.30.16	1.4	Commerce and advertising	No
Musiconomi	9.28.17	5.3	Content mgmt.	Yes	Ethbits	5.13.17	1.3	Trading and exchanges	Yes
Exscudo	5.31.17	5.3	Payments	No	Databits	2.28.17	1.1	Video games and VR	No
Betmaster	12.31.17	5.2	Gambling	No	Adelphoi	5.31.17	1.0	Finance	No
EncrypGen	7.18.17	n/a	Drugs and healthcare	No	Time New Bank	11.24.17	n/a	Finance	No
Playkey	11.30.17	n/a	Video games and VR	No	Chronologic	9.4.17	n/a	Blockchain Infra.	No
Protos	12.15.17	n/a	Content mgmt.	No	Giga Watt Token	7.31.17	n/a	Crypto mining	No
Matryx	11.20.17	n/a	Video games and VR	No	Sphre AIR	6.30.17	n/a	Identity and reputation	No
Dovu	10.17.17	n/a	Marketplaces	Yes	Nitro	12.26.17	n/a	Video games and VR	No
Vezt	12.3.17	n/a	Art and music	No	Block Array	1.8.18	n/a	Data analytics	Yes
Loopring	8.16.17	n/a	Trading and exchanges	No	SingularDTV	10.29.16	n/a	Events and entertainment	No
MyBit Token	8.26.17	n/a	Finance	No	CyberMiles	12.3.17	n/a	Commerce and advertising	No
Propy	9.15.17	n/a	Real estate	No	Agrello	8.17.17	n/a	Legal	No
Fusion	2.10.18	n/a	Finance	Yes	LATOKEN	10.10.17	n/a	Finance	No
Viberate	9.3.17	n/a	Art and music	Yes	EncryptoTel	5.11.17	n/a	Communications	No
ChainLink	9.19.17	n/a	Blockchain Infra.	No	MediBloc	12.15.17	n/a	Drugs and healthcare	No
Pally	12.13.17	n/a	Tourism	No	ICOS	9.15.17	n/a	Finance	No
Clout	12.17.17	n/a	Media	No	SwissBorg	1.10.18	n/a	Finance	No
Rentberry	2.28.18	n/a	Real estate	No	DomRaider	10.9.17	n/a	Payments	No
AirSwap	10.10.17	n/a	Trading and exchanges	No	Storj	6.7.17	n/a	Data storage	Yes
Request Network	10.15.17	n/a	Payments	No	LOCIcoin	12.31.17	n/a	Search	No
Veritaseum	5.26.17	n/a	Finance	No	Hive	7.31.17	n/a	Finance	No
Cashaa	12.6.17	n/a	Finance	No	Lamden	1.4.18	n/a	Blockchain Infra.	No
CanYa	12.27.17	n/a	Marketplaces	No	ZenGold	5.26.17	n/a	Commodities	No
CoinDash	7.17.17	n/a	Trading and exchanges	No	InvestFeed	8.7.17	n/a	Trading and exchanges	No
Telcoin	12.30.17	n/a	Payments	No	ATBCoin	7.12.17	n/a	Payments	No
Cobinhood	10.22.17	n/a	Trading and exchanges	No	Tokenbox	11.28.17	n/a	Finance	No
Mothership	7.28.17	n/a	Trading and exchanges	Yes	LeadCoin	3.1.18	n/a	Commerce and advertising	No
Nimiq	7.28.17	n/a	Blockchain Infra.	No	DreamTeam	12.14.17	n/a	Video games and VR	No
Achain	7.7.17	n/a	Blockchain Infra.	No	Publica	12.1.17	n/a	Content mgmt.	No
Komodo	11.20.16	n/a	Blockchain Infra.	No	Genaro Network	11.30.17	n/a	Blockchain Infra.	No
Seratio Project	10.31.17	n/a	Charity	No	Aeron	10.30.17	n/a	Tourism	No
CoinStarter	2.17.18	n/a	Finance	No	Ecobit	6.15.17	n/a	Agriculture	No
QASH	11.8.17	n/a	Finance	No	Sphre AIR	6.30.17	n/a	Identity and reputation	No
Gameflip	1.29.18	n/a	Video games and VR	No	Nitro	12.26.17	n/a	Video games and VR	No
Corion Platform	8.27.17	n/a	Blockchain Infra.	No	Block Array	1.8.18	n/a	Data analytics	Yes
Universa	12.8.17	n/a	Blockchain Infra.	No	SingularDTV	10.29.16	n/a	Events and entertainment	No
Moria	2.25.18	n/a	Commodities	No	CyberMiles	12.3.17	n/a	Commerce and advertising	No
Chaintrade	12.16.17	n/a	Commodities	No	Agrello	8.17.17	n/a	Legal	No
Leverj	12.6.17	n/a	Trading and exchanges	No	LATOKEN	10.10.17	n/a	Finance	No
DAO.Casino	7.21.17	n/a	Gambling	No	EncryptoTel	5.11.17	n/a	Communications	No
Bitquence	7.16.17	n/a	Finance	No	MediBloc	12.15.17	n/a	Drugs and healthcare	No
HADE	1.26.18	n/a	Data analytics	No	ICOS	9.15.17	n/a	Finance	No
Banca	2.26.18	n/a	Marketplaces	No	SwissBorg	1.10.18	n/a	Finance	No
Iungo	1.31.18	n/a	Communications	No	DomRaider	10.9.17	n/a	Payments	No
adToken	6.26.17	n/a	Commerce and advertising	No	Storj	6.7.17	n/a	Data storage	Yes
CommerceBlock	12.19.17	n/a	Finance	No	LOCIcoin	12.31.17	n/a	Search	No
Paragon	10.15.17	n/a	Real estate	No	Hive	7.31.17	n/a	Finance	No
REALT	10.31.17	n/a	Commerce and advertising	No	Lamden	1.4.18	n/a	Blockchain Infra.	No
Gifto	12.14.17	n/a	Payments	Yes	ZenGold	5.26.17	n/a	Commodities	No
bitJob	10.12.17	n/a	Marketplaces	No	bitJob	10.12.17	n/a	Marketplaces	No
Crederoom	11.13.17	n/a	Finance	No	InvestFeed	8.7.17	n/a	Trading and exchanges	No
iDice	6.26.17	n/a	Gambling	No	ATBCoin	7.12.17	n/a	Payments	No

The table lists all 306 ICOs in the sample, ordered by the amount raised, in descending order. The total amount raised is specified in millions of US dollars. Where the total is listed as n/a, secondary sources indicated a total exceeding $1 m but we were unable to establish the exact amount raised in the presale and/or crowdsale using primary sources. The end date is specified in the format month/day/year. The column Inv. sample indicates which of the ICOs are part of the “investor sample”

## Appendix 2: Definition of variables

See Table 10.

**Table 10:** Definition of variables

Variable name	Type	Definition	Source(s)
Amount raised in crowdsale	Continuous	Total amount of funds (in US dollars) raised during the ICO’s crowdsale stage. Where possible, the total is calculated by multiplying the amounts of cryptocurrencies received by their closing price on the last day of the ICO. Where amounts in cryptocurrency are unavailable, the US dollar figures disclosed by the ICO’s promoter are used. If the ICO conducts a presale *without* any effective restrictions (such as participation by invitation only, or a minimum investment requirement above USD 5000) on participants, funds raised during the presale are counted toward the crowdsale	Company website, ICO documentation, social media
Amount raised in presale	Continuous	Total amount of funds (in US dollars) raised during the ICO’s presale stage. Where possible, the total is calculated by multiplying the amounts of cryptocurrencies received by their closing price on the last day of the ICO. Where amounts in cryptocurrency are unavailable, the US dollar figures disclosed by the ICO’s promoter are used	Company website, ICO documentation, social media
Business model available	Indicator	The documentation details the market opportunity the product financed by the ICO addresses and lays out how the company will eventually earn money	Company website, ICO documentation, social media
Celebrity endorsement	Indicator	The ICO is being promoted by a popular entertainment or sports personality on social media	Company website, social media
Crowdsale is auction	Indicator	The token price for crowdsale investors depends on the total amount of funds raised during the crowdsale	Company website, ICO documentation, social media
Crowdsale max. discount	Continuous	Maximum discount given to (usually large or early) investors during the crowdsale stage. Calculated as Crowdsale max. discount = (maximum crowdsale price − minimum crowdsale price)/maximum crowdsale price	Company website, ICO documentation, social media
Development road map available	Indicator	The documentation contains a road map with dates and milestones for the development and commercialization of the product	Company website, ICO documentation, social media
Experienced team	Indicator	The founding team has an average of at least ten years of experience in technology, management or entrepreneurship	Company website, ICO documentation, social media
Financial advisor disclosed	Indicator	The financial/blockchain expert (either a company or an individual) who advised the company in arranging its ICO is disclosed	Company website, ICO documentation, social media
Funding milestones	Indicator	The terms of funding lay out binding milestones (e.g., development of a working prototype) that need to be met in order for the funds raised in the ICO to be released to the firm	Company website, ICO documentation, social media
Fundraiser has maximum (“hard cap”)	Indicator	There is a maximum number of tokens the company will sell in its ICO	Company website, ICO documentation, social media
Fundraiser has minimum (“soft cap”)	Indicator	There is a minimum number of tokens to be sold or money to be raised for the ICO to be considered a success	Company website, ICO documentation, social media
Has a presale	Indicator	The ICO has a dedicated presale stage reserved for large investors. Zero if the presale has no minimum investment requirement	Company website, ICO documentation, social media
Has VC backing	Indicator	The company has received funding from a venture capitalist, in exchange for an equity stake or tokens, prior or during the ICO	Company website, ICO documentation, social media, Crunchbase
High-quality advisory team	Indicator	Advisory team is of high quality, i.e., mostly composed of individuals with significant experience as entrepreneurs, executives, venture investors or academics	Company website, ICO documentation, social media
Independent custodian for ICO funds	Indicator	The funds raised in the ICO are held by an independent third party, e.g., a Swiss foundation where the majority of the foundation board is composed of individuals not presently in a business relationship with the promoter of the ICO	Company website, ICO documentation, social media, commercial registers
Investors from other jurisdictions excluded	Indicator	Investors from jurisdictions other than the US are not allowed to participate in the ICO (most commonly countries that have banned ICOs such as China and South Korea and countries on the OFAC sanctions list)	Company website, ICO documentation, social media
Investors have governance rights	Indicator	Token holders have a right to vote on investment, business or governance decisions. Includes advisory votes	Company website, ICO documentation, social media
Is a security	Indicator	The token likely qualifies as a financial security. Most commonly because it pays interest or dividends, because the issuing firm commits to buybacks using the firm’s net income or because the token represents a physical asset or a share in an investment fund	Company website, ICO documentation
Is a utility token	Indicator	The token is intended to be used primarily for consumption of a product or services and does not generate cash distributions to holders	Company website, ICO documentation
Is cryptographic token	Indicator	The ICO takes the form of a smart contract on an existing blockchain (e.g., Ethereum, Waves, Qtum, Nxt)	Company website, ICO documentation
Is currency or general-purpose blockchain		The token is intended to be used primarily as a currency, replacing traditional fiat money, or as the unit of account for a new general-purpose blockchain able to execute smart contracts	Company website, ICO documentation
Issuer has customers for product	Indicator	The product or service underlying the ICO has users (regardless of whether they pay for the service or not)	Company website, ICO documentation, social media
KYC/AML procedure	Indicator	The ICO’s promoter required participants to identify themselves by submitting personal documents such as a passport copy, utility bills, etc.	Company website, ICO documentation, social media
Legal advisor disclosed	Indicator	The legal expert (either a company or an individual) who advised the company in arranging its ICO is disclosed	Company website, ICO documentation, social media
Legal form and jurisdiction known	Indicator	Type of legal entity (e.g., limited liability company or joint-stock corporation) and jurisdiction of incorporation of the entity conducting the ICO are disclosed	Company website, ICO documentation, commercial registers
Legal form is foundation	Indicator	The issuing entity is a not-for-profit foundation (typically incorporated in Switzerland or Liechtenstein)	Company website, ICO documentation, commercial registers
Legal entity is corporation	Indicator	The issuing entity is a joint-stock corporation or its equivalent in non-US jurisdictions	Company website, ICO documentation, commercial registers
Legal entity is LLC	Indicator	The issuing entity is a limited liability corporation (LLC) or limited liability partnership (LLP) or their equivalent in non-US jurisdictions	Company website, ICO documentation, commercial registers
Length of ICO (calendar days, actual)	Discrete	Actual length of the crowdsale period in number of days	Company website, ICO documentation, social media
Length of ICO (calendar days, planned)	Discrete	Planned maximum length of the *crowdsale* period in number of days	Company website, ICO documentation, social media
Lockup period unsold tokens	Continuous	Weighted average of the period over which unsold tokens are locked up (i.e., cannot be sold). Equals zero if the tokens are not locked up	Company website, ICO documentation, social media
Percentage of hard cap raised	Continuous	Fraction of the maximum amount the company manages to raise during its ICO	Company website, ICO documentation, social media
Postal address known	Indicator	Physical postal address of the ICO promoter’s headquarters is known	Company website, ICO documentation, commercial registers
Presale discount	Continuous	Presale discount over the crowdsale “list price,” based on original price quotes in cryptocurrencies where available for both presale and crowdsale, otherwise based on converted US dollar prices Presale discount = (maximum crowdsale price – minimum presale price)/maximum crowdsale price	Company website, ICO documentation, social media
Presale lockup period (weighted avg.)	Continuous	Lockup period of tokens sold during the presale stage. Where tokens are subject to a vesting schedule or only part of the tokens is locked up, we track the weighted average maturity of all tokens sold at the presale stage. Where different fractions of presold tokens are subject to different lockup periods, but the size of those fractions is unclear, we calculate the weighted average maturity based on the minimum lockup period	Company website, ICO documentation, social media
Presale tokens locked up	Indicator	Tokens sold during the presale stage cannot be sold for a certain period of time	Company website, ICO documentation, social media
Product can be tried out	Indicator	Prospective investors can try the product or prototype	Company website, ICO documentation, social media
Product or prototype developed	Indicator	The product for which funding is being raised or an early “alpha” or “beta” version of it has been developed	Company website, ICO documentation, social media
Project code available	Indicator	The company provides original source code for the project it is raising money for on Github as of the first day of the ICO	Github
Qualified investors only	Indicator	Only investors with accredited investor status or equivalent are allowed to participate in the ICO	Company website, ICO documentation, social media
Registered in offshore financial center	Indicator	The jurisdiction of incorporation is an offshore financial center as per the definition of the International Monetary Fund	Company website, ICO documentation, commercial registers
Simple agreement for future tokens (SAFT)	Indicator	The ICO employs a “Simple Agreement for Future Tokens” (SAFT) under which tokens are only issued once the platform on which they can be used has been released	Company website, ICO documentation
Smart contract code available	Indicator	If the token sold during the ICO takes the form of a smart contract on another blockchain, is the source code for the smart contract available on Github prior to the ICO?	Github
Team business background missing	Indicator	Insufficient information to determine the value of the variable “team member with business background”	Company website, ICO documentation, social media
Team experience missing	Indicator	Insufficient information to determine the value of the variable “experienced team”	Company website, ICO documentation, social media
Team lockup period (weighted avg.)	Continuous	Weighted average maturity of the tokens under control of the issuing company and the founding team. Includes all the tokens also included in “Token share team (ex ante)”	Company website, ICO documentation, social media
Team member with business background	Indicator	At least one of the team members has significant experience in entrepreneurship, consulting or management	Company website, ICO documentation, social media
Team size	Discrete	Number of full-time team member at the time of the ICO, excluding advisors and contractors	Company website, ICO documentation, social media
Team tokens locked up	Indicator	Some fraction of the tokens held by the issuing company and/or the founding team are subject to a vesting schedule	Company website, ICO documentation, social media
Time to listing (calendar days)	Discrete	Number of days between the last day of the crowdsale period and the first day for which a closing price is listed on coinmarketcap	Company website, ICO documentation, social media, coinmarketcap
Token share crowdsale investors (ex ante)	Continuous	Fraction of total token supply allocated to crowdsale investors following the crowdsale, assuming the crowdsale sells out	Company website, ICO documentation, social media
Token share crowdsale investors (ex post)	Continuous	Fraction of tokens held by crowdsale investors after the crowdsale, after all tokens have been distributed and unsold tokens destroyed or allocated to the issuer	Company website, ICO documentation, social media
Token share presale investors (ex ante)	Continuous	Fraction of total token supply allocated to presale investors following the crowdsale, assuming the crowdsale sells out	Company website, ICO documentation, social media
Token share producers/miners (ex ante)	Continuous	Fraction of total token supply reserved for “miners” or producers on the platform following the crowdsale, assuming the crowdsale sells out	Company website, ICO documentation, social media
Token share team (ex ante)	Continuous	Fraction of total token supply under control of the issuing firm and the founding team following the crowdsale, assuming the crowdsale sells out. Includes all tokens under the control of the firm, including tokens reserved promotional activities, “bounties” (compensation for promotional activities), compensation of suppliers, employees and advisors and any other residual categories	Company website, ICO documentation, social media
Token supply is fixed	Indicator	The total number of tokens stays fixed indefinitely, as opposed to tokens that allow for inflation or the creation of additional tokens under certain circumstances	Company website, ICO documentation, social media
Total amount raised	Continuous	Total amount of funds (in US dollars) raised during the ICO. Includes funds raised during crowdsale and presale. Where possible, the total is calculated by multiplying the amounts of cryptocurrencies received by their closing price on the last day of the ICO. Where amounts in cryptocurrency are unavailable, the US dollar figures disclosed by the ICO’s promoter are used	Company website, ICO documentation, social media
Unknown or low-quality advisors	Indicator	Advisory team is either unknown or of low quality (i.e., mostly composed of “crypto evangelists,” celebrities or similar)	Company website, ICO documentation, social media
Unsold tokens “burnt” or proportional allocation	Indicator	Unsold tokens are either destroyed or the token allocation is done proportionally (e.g., the team receives 20% of all tokens created following the crowdsale, regardless of its result)	Company website, ICO documentation, social media
Unsold tokens kept by issuer	Indicator	The issuer retains unsold tokens, either for future token sales or to be used for a different purpose	Company website, ICO documentation, social media
US retail investors excluded	Indicator	Non-accredited investors from the United States are not allowed to participate in the ICO	Company website, ICO documentation, social media
Use of proceeds disclosed in detail	Indicator	The issuer provides a detailed breakdown for the use funds raised during the ICO (e.g., X software developers at Y dollars and hour are required to do Z hours of work to complete the product)	Company website, ICO documentation, social media
Use of proceeds mentioned	Indicator	The issuer provides a rough breakdown for the use of funds raised during the ICO (e.g., 40% product development, 10% legal, 50% marketing)	Company website, ICO documentation, social media
Utility token enables decentralization	Indicator	The funds raised in the ICO are used to develop a decentralized platform on which buyers and sellers of a particular service or product engage in market-based interaction, as opposed to the company conducting the ICO being or becoming the sole provider of the service or product	Company website, ICO documentation, social media
Whitepaper page count	Discrete	Number of pages in the white paper document	ICO documentation
Years since foundation	Discrete	Years since the founding team started working on the project for which the ICO is being conducted. Where unavailable, the date of incorporation from the commercial register is used. Rounded to the nearest integer	Company website, ICO documentation, social media, commercial registers

## Appendix 3: Additional descriptive statistics

See Table 11.

**Table 11:** Additional descriptive statistics

	Mean	Median	Min	Max	SD	*N*
*Panel A: ICO attributes*						
Is currency or general-purpose blockchain	0.16	0.00	0.00	1.00	0.36	306
Is a utility token	0.61	1.00	0.00	1.00	0.49	306
Length of crowdsale (calendar days, actual)	28.45	29.50	1.00	148.00	22.43	306
Length of crowdsale (calendar days, planned)	31.92	31.00	1.00	148.00	21.66	303
Time to listing (calendar days)	19.92	13.00	− 319.00	222.0	39.75	275
Crowdsale is auction	0.11	0.00	0.00	1.00	0.32	306
Token supply is fixed	0.89	1.00	0.00	1.00	0.31	306
Token share crowdsale investors (ex post)	0.42	0.40	0.00	1.00	0.27	227
Unsold tokens kept by issuer	0.45	0.00	0.00	1.00	0.50	306
Lockup period unsold tokens (years)	0.39	0.00	0.00	10.00	1.22	138
Smart contract code available	0.67	1.00	0.00	1.00	0.47	278
Utility token enables decentralization	0.75	1.00	0.00	1.00	0.44	186
Financial advisor disclosed	0.19	0.00	0.00	1.00	0.39	306
Simple agreement for future tokens (SAFT)	0.03	0.00	0.00	1.00	0.16	306
*Panel B: Company attributes*						
Whitepaper page count	30.54	27.00	0.00	127.00	17.40	302
Business model available	0.67	1.00	0.00	1.00	0.47	306
Project code available	0.29	0.00	0.00	1.00	0.45	306
Development road map available	0.79	1.00	0.00	1.00	0.41	306
Issuer has customers for product	0.20	0.00	0.00	1.00	0.40	306
Use of proceeds disclosed in detail	0.05	0.00	0.00	1.00	0.22	306
Experienced team	0.58	1.00	0.00	1.00	0.50	306
Product can be tried out	0.40	0.00	0.00	1.00	0.49	306
Team size	11.46	9.00	2.00	80.00	9.20	282
Team member with business background	0.56	1.00	0.00	1.00	0.50	306
Years since foundation	1.60	1.00	0.00	16.00	2.06	306
Unknown or low-quality advisors	0.32	0.00	0.00	1.00	0.47	306
Celebrity endorsement	0.04	0.00	0.00	1.00	0.19	306
Postal address known	0.70	1.00	0.00	1.00	0.46	306
Legal entity is foundation	0.07	0.00	0.00	1.00	0.26	306

The table shows additional summary statistics for a hand-collected sample of 306 ICOs that took place between March 2016 and March 2018. All variables are defined in “Appendix 2.” The time to listing is negative if the token starts trading on an exchange before the end of the crowdsale

## Footnotes

1 Also see PwC Switzerland, 2019, 5th ICO/STO Report. https://www.pwc.ch/en/publications/2019/ch-PwC-Strategy&-ICO-Report-Summer-2019.pdf.

2 See, for example, Gandal et al. (2018), Xu and Livshits (2018) and Li et al. (2018).

3 In equity markets, short selling costs are high for small, illiquid stocks with low institutional ownership and a large amount of disagreement about the firm’s intrinsic value between investors (D’Avolio 2002). Most ICO tokens have a low market capitalization compared to the average publicly listed firm, have low institutional ownership and feature large differences in opinions between investors. With the exception of Bitcoin, there are also no exchange traded derivatives for cryptoassets on major trading venues. Finally, short selling is risky if fundamental values and prices take a long time to converge, because the arbitrageur might suffer reputational damage and liquidity shortfalls before profits are eventually realized (Shleifer and Vishny 1997).

4 E.g., Adhami et al. (2018), Amsden and Schweizer (2019), Benedetti and Kostovetsky (2018), Boreiko and Sahdev (2018), Bourveau et al. (2019), Jong et al. (2019), Deng and Lee (2018), Dittmar and Wu (2019), Fisch (2019), Howell et al. (2019), Lee et al. (2018), Lyandres and Palazzo (2020) and Momtaz (2020).

5 Lyandres and Palazzo (2020) focus mainly on the determinants of ICO success, but also collect data on the evolution of the number of wallets after an ICO starts trading and correlate it with trading volume. Fisch et al. (2018) provide survey evidence on the investment motives for a sample of 500 ICO investors.

6 By construction, our sample features higher averages for funds raised and fundraising success.

7 Many ICOs only allow contributions in cryptocurrencies, primarily Ethereum and Bitcoin. Because the dollar value of such cryptocurrencies is volatile, we collect the amounts of funding raised in cryptocurrencies where available. We then calculate the value of total funding raised, in US dollars, using closing prices on the last day of the contribution period. We only rely on totals in US dollars disclosed by issuers where the detailed breakdown into cryptocurrencies is not available.

8 The raw primary market database can be downloaded free of charge from Zenodo. 10.5281/zenodo.4034258

9 We base our calculations on prices instead of total returns because we do not observe interest and dividend payments made by the 22% of the sample composed of security tokens. For robustness, we repeat—but do not show—all calculations on the subsample of ICOs that issue utility tokens and that therefore cannot make any cash distributions. The results closely resemble those of the full sample, implying that security tokens do not affect the fundamental conclusions of our analysis.

10 We thank Evgeny Medvedev for providing computer code to export data from the Ethereum blockchain (see https://github.com/medvedev1088/ethereum-etl).

11 We only require 98 out of 100 transactions because sometimes a token is mintable, but the entirety of the token distribution is first minted to (i.e., transferred from the zero address) one or more addresses controlled by the ICO’s organizers and then redistributed from those secondary addresses to investors. If no address is at least 98 times the source of initial distribution, we say that we cannot identify the origin and do not analyze the token further.

12 We refer the reader to Amsden and Schweizer (2019) and Howell et al. (2019) for more detailed descriptive statistics of the ICO market. “Appendix 3” features more extensive summary statistics on our sample.

13 Some sources refer to assets issued on a standalone blockchain as cryptocurrencies and to those implemented through smart contracts as cryptographic tokens. In the remainder of this document, we will refer to all cryptographic assets sold in ICOs as *tokens,* regardless of their technical implementation.

14 In very few cases, the presale discount is negative. This can happen if the ICO promoter lowers token prices before the crowdsale because of an appreciation against the US dollar in the cryptocurrency accepted in the ICO.

15 According to data from etherscan.io, the average fee for sending Ether from one address to another was $0.13 between the start of the first and the end of the last ICO in our sample. Fees for sending ERC20 tokens depend on the token and are higher than those for sending Ether, but they are of the same order of magnitude.

16 The mean self-reported number of investors is 5555.6, and the mean of our estimated number is 5169.4 (*n *= 18, *t *= 1.08).

17 The mean of the estimated number of contributors is 5656.8 (*n *= 58) for ICOs with a KYC policy and 3310.0 (*n *= 40) for ICOs without one, with a *t*-stat of − 1.73 for the difference.

18 Wong et al. (2009) formally define angels as those that are “accredited investors” according to SEC Regulation D, Rule 501. Rule 501 states that accredited investors must have a net worth of over $1 m or annual income of over $200,000.

19 Table 1 shows, however, that 75% of the total contribution come from the crowdsale event, so that most of the money raised in an ICO comes from crowdsale investors.

20 The regression includes the ICO’s hard cap. Therefore, the coefficient estimate for the fraction of tokens retained by founders represents the effect on the number of investors given a constant number of tokens offered for sale. The correlation between the founder allocation and the number of investors is therefore not purely mechanical.

21 Using a different methodology, Boreiko and Risteski (2019) find that 24.3% of addresses contribute to more than one ICO on Ethereum.

22 For this analysis, we only retain tokens with a mean daily trading volume of at least $1000 and mean market cap of at least $100,000 during their first 2 weeks of trading. This filter leaves us with a sample of 449 ERC20 tokens.

23 The average daily trading volume for the median ICO is $193,976 over the first 90 days following the ICO. Therefore, secondary markets seem to be liquid enough to allow investors to liquidate their positions relatively easily should they wish to do so.

24 Cheng et al. (2019) show that publicly listed firms experience positive stock market returns when they announce that they are going to use blockchain technology, although these companies are not experts in blockchain technology and do not offer any specifics of their projects. In this case, however, the stock market returns reverse shortly after, possibly because it is easier to take short positions in the large and mature stocks of their sample.

25 Krigman et al. (1999) show that large and informed traders flip the IPO allocations that perform the worst in the future.

26 Greene (2004) finds that the incidental parameter problem, which commonly affects nonlinear regression models with fixed effects, usually does not impact the coefficient estimates in Tobit models.

27 A caveat with the result that investors experience positive returns on average is that there is evidence of price manipulation in cryptocurrency markets (Gandal et al. 2018; Xu and Livshits 2018; Li et al. 2018). The literature shows that manipulative trading in cryptocurrencies can lead to inflated prices. Gandal et al. (2018) in particular show that these inflated prices can persist for prolonged periods. If the ICO tokens we study are subject to such manipulation, it is possible that the positive returns we find will not last beyond our sample horizon.

28 We have also re-estimated the regressions of Table 8 by including country fixed effects, with quantitatively and qualitatively similar results.
